# Landscape of toxin-neutralizing therapeutics for snakebite envenoming (2015–2022): Setting the stage for an R&D agenda

**DOI:** 10.1371/journal.pntd.0012052

**Published:** 2024-03-26

**Authors:** Juliette Borri, José María Gutiérrez, Cecilie Knudsen, Abdulrazaq G. Habib, Maya Goldstein, Andrew Tuttle

**Affiliations:** 1 Policy Cures Research, Sydney, New South Wales, Australia; 2 Instituto Clodomiro Picado, Facultad de Microbiología, Universidad de Costa Rica, San José, Costa Rica; 3 VenomAid Diagnostics, Lyngby, Denmark; 4 Infectious and Tropical Diseases Unit, Department of Medicine, Bayero University, Kano, Nigeria; Fundação de Medicina Tropical Doutor Heitor Vieira Dourado, BRAZIL

## Abstract

**Background:**

Progress in snakebite envenoming (SBE) therapeutics has suffered from a critical lack of data on the research and development (R&D) landscape. A database characterising this information would be a powerful tool for coordinating and accelerating SBE R&D. To address this need, we aimed to identify and categorise all active investigational candidates in development for SBE and all available or marketed products.

**Methodology/Principal findings:**

In this landscape study, publicly available data and literature were reviewed to canvas the state of the SBE therapeutics market and research pipeline by identifying, characterising, and validating all investigational drug and biologic candidates with direct action on snake venom toxins, and all products available or marketed from 2015 to 2022. We identified 127 marketed products and 196 candidates in the pipeline, describing a very homogenous market of similar but geographically bespoke products and a diverse but immature pipeline, as most investigational candidates are at an early stage of development, with only eight candidates in clinical development.

**Conclusions/Significance:**

Further investment and research is needed to address the shortfalls in products already on the market and to accelerate R&D for new therapeutics. This should be accompanied by efforts to converge on shared priorities and reshape the current SBE R&D ecosystem to ensure translation of innovation and access.

## Author summary

Snakebite envenoming (SBE) is a neglected tropical disease that exerts a high impact worldwide, especially in sub-Saharan Africa, Asia, and Latin America. Animal-derived antivenoms constitute the only specific, scientifically validated treatment for envenomings, although new therapeutic alternatives are being pursued. Despite the relevance of this disease, there is a critical lack of data on the research and development landscape in antivenoms and novel therapeutics. This study describes a thorough search of the state of SBE therapeutic market and research pipeline. A total of 127 marketed products and 196 candidates were identified. The current antivenom landscape is characterised by a homogeneous market of similar but geographically bespoke products needing improvement. The R&D pipeline analysis component revealed a diverse but immature picture since only eight candidates are in clinical development. This article interprets the findings of the study to provide an initial list of priority areas that sets the stage for further development of an R&D agenda to ensure translation of innovation to reduce the impact of SBE.

## Introduction

Snakebite envenoming (SBE) is a complex and neglected global health challenge that kills and injures hundreds of thousands of people every year, especially in impoverished rural communities in Africa, Asia, and Latin America [1]. In recent years, a concerted effort to raise the profile of SBE and stimulate investment in R&D has yielded positive outcomes. This includes the official inclusion of SBE in the World Health Organization (WHO) Neglected Tropical Disease (NTD) portfolio in 2017, a resolution on SBE adopted by the World Health Assembly in 2018, and the launch of the WHO 2019–2030 roadmap to prevent and control SBE in 2019 [2,3]. Looking to R&D, the community has seen progress in the development of the WHO Target Product Profile for sub-Saharan African antivenoms [4], a WHO risk-benefit assessment program for antivenoms [5], and a set of global core outcome measurements for clinical trials on SBE [6]. As work in this space progresses, there is a continued and growing need to provide the sector with data and information to guide funding decisions and research agendas.

SBE R&D funding has benefitted from four years of consecutive growth from 2018–2021, with more funding invested in that period than in the decade from 2007–2017 [7]. SBE has been one of the only WHO NTDs to see increased funding, with many of the other diseases experiencing a decline or plateau in recent years [8]. Despite this positive trend, there is concern around the sustainability of funding and how it will impact translating research into real-word solutions. Funding for SBE R&D is concentrated in a select number of both private and public backers and is heavily project-linked. As a result, shifts in individual funders and projects could potentially severely set back the SBE R&D landscape. Much of the growth in the sector has been associated with UK public and philanthropic funders, specifically Wellcome’s £80 million programme [8], which has facilitated important basic and early-stage research and resolved several barriers to generating knowledge. However, it is due to end in 2026. Another top funder of SBE R&D has been the US Department of Defense (DOD), which currently only invests in one product developer for one drug, with future investment in SBE research not guaranteed. Since 2018, the collective share of total funding from Wellcome and the US DOD has risen from a low of 7% in 2019, to 71% in 2022 [8]. Having the bulk of SBE R&D funding concentrated in two funders is risky for the sector, even more so when these are not sustainable streams. To date, most of the funding has also focused on early-stage R&D, highlighting a lack of interest and investment in late-stage R&D, as well as implementation and operational research, manufacturing, and access activities. Actions need to be taken to ensure the translation of research from basic and early-stage into clinical development will be resourced and to sustain the momentum the sector has garnered to deliver new products to those that need them the most.

Essential to achieving this will be overcoming certain structural issues in the SBE R&D ecosystem to guarantee a diversified set of investors and portfolios. The solutions to these challenges are not yet clear but require robust information about the SBE R&D landscape–data which has been previously missing, incomplete, or out-of-date. Here we summarise the results of a recent, comprehensive landscape analysis conducted to better understand the state of the research pipeline for novel therapeutics and the antivenom market for SBE. It builds on a series of foundational reviews on antivenoms [9–11], novel and repurposed drugs, and biologics as potential SBE therapeutics [12–17] and diagnostics [18]. The study provides critical data to begin asking and answering key questions about the future of the SBE R&D landscape.

Several reviews on various aspects of the SBE R&D landscape have been undertaken in the last decade. These include reviews of the clinical status of available antivenoms in sub-Saharan Africa; systematic reviews of clinical outcome measures for SBE in randomised control trials; the SBE diagnostic pipeline; small molecule therapies (SMTs) and repurposed drugs; plant polyphenols; and traditional medicine as potential SBE therapeutics. Also, prior to this study, several comprehensive overviews of novel small molecules and biologics with snake toxin neutralisation abilities have been published.

While previous reviews have been critical sources of information on the therapeutics pipeline and market, the last comprehensive landscaping was done in 2018, and only for new therapeutics, not current therapies. The recent years of advancement in SBE R&D funding warrant updating the community’s knowledge of the full spectrum of candidates under development and products currently available or in use (from 2015–2022). To this end, a database was created based on interactive, open-source information of candidate and product profiles. The database includes 127 available products and 196 investigational candidates (in various stages of development) and provides a unique, unified data source with standardised information, contributing to improved transparency and clarity of the landscape.

By presenting insight into the state of the current SBE R&D landscape for new and existing therapeutics, our results encourage further investment and research into improving products on the market as well as maintaining and accelerating development for investigational candidates to ensure that more products not only reach clinical development but can exit the pipeline. This database contributes to a growing body of evidence on SBE that will shape the R&D landscape and inform decisions on how research is prioritised.

## Methods

### Scope

We established a comprehensive database profiling (a) all drugs and biologics registered and/or available for the treatment of SBE (with direct action on toxins) since 2015 (‘products’) and (b) all drugs and biologics investigated as potential SBE therapeutics (with direct action on toxins) since 2015 (‘investigational candidates’). Specific exclusions were: adjunct and supportive therapies that only modify immune responses and clinical manifestations caused by snake venom toxins, including products related to wound and adverse reaction management; devices, diagnostics, and other non-medicine-related biomedical products with an indication for SBE; and basic and fundamental research not geared towards product development. Further scope considerations are described in detail in S1 Text.

### Development of a medicines database

**Fig 1:**
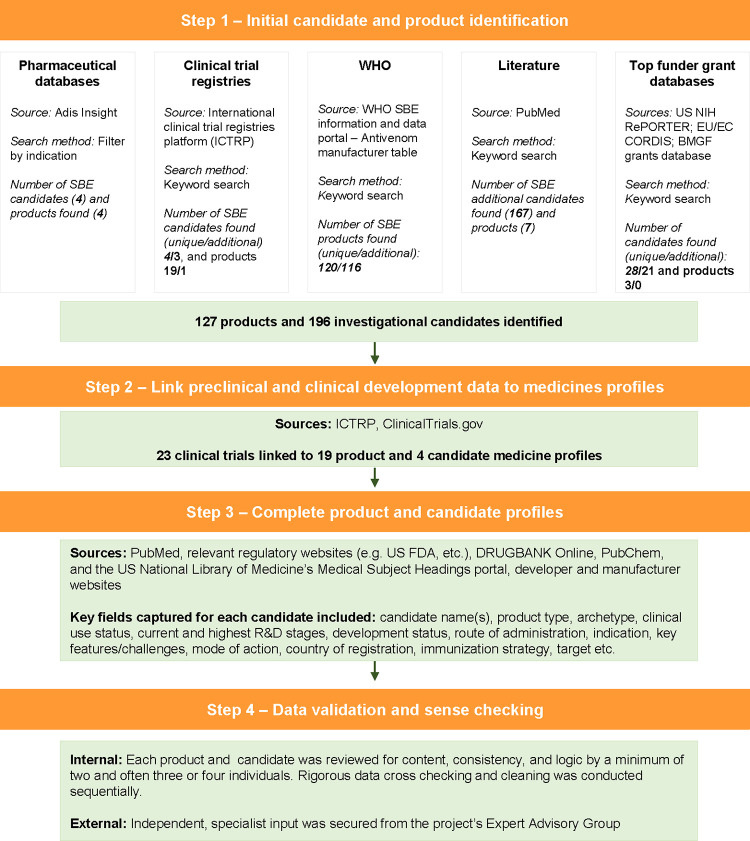
Methodology flow chart (adapted from McDougall et al., 2022) [19].

The complete methodology of how this database was developed is described in detail in S1 Text. To summarise, we undertook a series of partially sequential, partly overlapping, but mutually reinforcing steps to develop a database of product and candidate profiles. These were: (a) identify and validate products and candidates that are or were in use or in development since 2015 through multiple sources; (b) collect information on the products’ and candidates’ preclinical and/or clinical development, and associated data; (c) research additional context around the products and candidates (e.g., paraspecificity) to build out individual multi-field profiles; (4) validate and sense check candidate profiles through independent, external reviews by experts in the field. Fig 1 outlines the methodology followed in the study.

For the initial candidate and product identification, multiple sources were used to identify and retrieve information on individual entries. We searched Adis Insight for up-to-date information on relevant drugs and biologics investigated for SBE. The WHO Snakebite Information and Data portal, specifically the Antivenom and Manufacturers table, was searched to extract a list of marketed products with relevant information on species indication, manufacturers, and registration. Data was also extracted from the WHO International Clinical Trials Registry Platform (ICTRP), which served a dual function of uncovering additional products and/or candidates for inclusion that had not yet been identified, as well as capturing and linking clinical trial data to candidates and products marked for inclusion in the database. The G-FINDER R&D funding database, which includes funding from public, private and philanthropic organisations from HICs and LMICs, was reviewed for relevant grant descriptions. An overview of organisations that have participated in the G-FINDER survey over the years can be found on the online portal. Publicly available databases from three of the largest funders of medicines development were also datamined: the United States National Institutes of Health (US NIH)’s RePORTER; the European Commission’s CORDIS; and the Bill & Melinda Gates Foundation’s (BMGF) grants database (data supplied by the Foundation); as well as those of two major global funders of SBE therapeutics R&D with available online data: Wellcome’s grant funding and the US Department of Defense (DOD) via USASpending.gov. A literature review was also conducted using PubMed to validate already identified products and candidates as well as uncover new ones for inclusion. Product and candidate entries were not only reviewed and de-duplicated at first instance, but results from the different data sources were then combined and again de-duplicated to ensure maximum coverage, while only unique entries were included.

Much of the information needed to complete profiles were provided through Steps (a) and (b). In addition, we conducted desk-based research to source greater detail and context e.g., developer websites, grant descriptions, and regulatory documents. Following database completion, a series of internal and external independent reviews were undertaken to clean, cross-check and validate the data. We sought independent input from members of the project’s external expert advisory group. The entire database was reviewed to validate entries or identify missing candidates or products; review essential standard labels; and for each, recommend corrections, improvements, or additional details. This methodology has been previously validated by Policy Cures Research through an earlier project mapping the landscape of the maternal health medicines pipeline [20].

## Results

**Fig 2:**
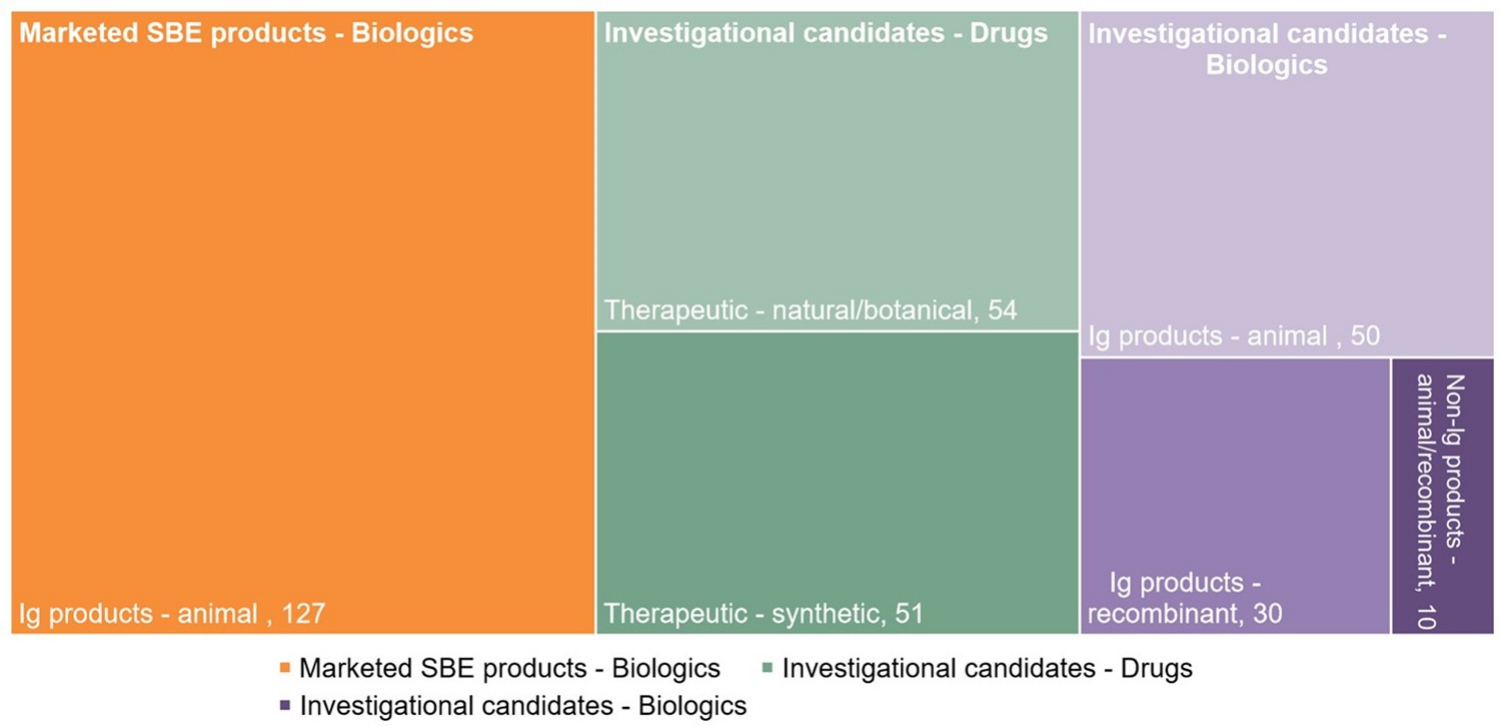
Summary of the available products and therapeutic candidates identified and included in the database of Policy Cures Research.

The result of this landscape analysis was the identification of 127 marketed and/or available products since 2015, along with 196 therapeutic candidates which have been actively investigated in the same period (Fig 2). The landscapes of products and candidates are strikingly different, highlighting major differences in product types, sub-types, and R&D stage.

### Marketed and/or available products

We identified an extremely homogenous market of 127 animal-plasma derived immunoglobulin-based antivenoms with no recombinant-based products and no drug treatments available for SBE. Nearly half of all products (61, 48%) are intended for use in the three regions with recognised high burdens of SBE: Southeast Asia (23, 18%), South America (20, 16%) and sub-Saharan Africa (18, 14%). Looking at the geography of manufacturers, we found that most products were produced for domestic needs within the manufacturer’s country or region. There was only one manufacturer in sub-Saharan Africa: South African Vaccine Producers. Almost all products manufactured for international use (largely for the African market) were from private companies, with just one public manufacturer, Instituto Clodomiro Picado from Costa Rica. This highlights a concerning concentration of manufacturing and supply power within international industry players for markets like Africa, where the need is great. In comparison Central and South America have a larger network of public laboratories that manufacture products for the region. Across Asia, there is a greater mix of public and private manufacturers mostly supplying the region–except for Indian manufacturers who export products to the African market.

Overall, most available antivenoms (111, 87%) had some evidence of being approved by either national or stringent regulatory approval authorities. For 16 products (13%) we could not ascertain clear regulatory approval despite their availability on the market (see S2 Text). Perhaps the most concerning finding was the limited publicly available preclinical and clinical evidence for products on the market. Just under half (57, 45%) of all products had available preclinical data of efficacy, and 29 (51%) of these also had available clinical trial data. Although a further 12 products had clinical data, but no publicly available preclinical data, the remaining 58 products–over a third in total–had no discernible preclinical data at all, despite this being the minimum standard for antivenoms to be approved for market use.

### Investigational candidates

Of the 196 investigational candidates identified, there was a relatively even split between R&D into novel drugs i.e., SMTs (105, 54%) and biologics (91, 46%) (S3 Text). This split is indicative of a broadening SBE R&D landscape. At the sub-product level, we saw a large variability in biologics, including novel whole immunoglobulins derived from animal models, pathology-specific antivenoms, as well as recombinant antibodies including human antibodies, camelid nanobodies, single-chain variable antibody fragments and others. For drug R&D, there was an even split between natural-derived (54, 51%) and synthetic SMT candidates (51, 49%). Synthetic SMTs include synthetic peptides, enzyme inhibitors, DNA aptamers, and nanoparticles, featuring some of the most clinically advanced candidates in the pipeline.

Overall, approximately 64% of SBE investigational candidates were new chemical or biological entities–largely driven by the nature of biologic research being so specific–while 36% were repurposed drugs, offering the advantage of existing safety data which could accelerate their development. The pipeline of investigational candidates remains immature however, with only eight candidates in clinical development (see Table 1). These include two candidates in Phase I; five in Phase II; and just one reported in Phase III. However, only four of these candidates appeared in active development (two SMTs and two traditional antivenoms), with the remaining (traditional antivenoms) having completed trials but with further progress unknown.

**Table 1:** Overview of the eight candidates in clinical development.

Candidate	Active component	Target	Phase	Active development	Developers/instigators
Novel F(ab’)2 antivenom	Serum-derived antibodies	*Daboia russelii siamensis*	Phase I	**Active**–Phase I registered 2020. Not yet recruiting.	Shanghai Serum Biotechnology Co Ltd
DMPS (Unithiol)	SMT	Snake venom metalloproteases	Phase I	**Active**–Phase I trial (“TRUE-1”) registered 2021. Recruiting.*	Liverpool School of Tropical Medicine
Novel ICP-AVRI-UOP Sri Lankan polyspecific antivenom	Serum-derived antibodies	*Daboia russelii*, *Echis carinatus*, *Hypnale hypnale*, *Naja naja*	Phase II/III	**Active**–Phase II/III trial registered 2016, updated in 2019. Recruitment pending 2019	Instituto Clodomiro Picado, University of Peradeniya, Animal Venom Research International
Novel freeze-dried trivalent antivenom (FDTAV)	Serum-derived antibodies	*Bothrops spp*., *Lachesis spp*., *Crotalus spp*.	Phase II	**Completed**–Phase IIb trial conducted 2003–2008, retrospectively registered 2017. Results published 2017. Further progress unknown	Instituto Butantan
Novel PNG taipan antivenom	Serum-derived antibodies	*Oxyuranus scutellatus*	Phase II	**Completed**–Phase II trial completed 2016. Results not published. Further progress unknown.	Instituto Clodomiro Picado, Australian Venom Research Unit
Novel bivalent snake antivenom	Serum-derived antibodies	*Daboia russelii*, *Echis carinatus*	Phase II	**Completed**–Phase II trial conducted in 2015. Results published 2017. Further progress unknown.	Antisnake venom and Antirabies Serology Laboratory, Sindh
Methyl-varespladib	SMT	Snake venom phospholipase A_2_s	Phase II	**Active**–Phase II trial (“BRAVO”) registered 2021, completed 2023 and awaiting results. Phase II trial (“BRAVIO”) registered 2023, recruiting*	Ophirex Inc.
Snake (Micrurus) North American immune F(ab’)2 equine	Serum-derived antibodies	*Micrurus fulvius*	Phase III	**Completed**–Phase III trial completed 2016. No official results published. Further progress unknown.	University of Arizona

* Updated for November 2023

## Discussion

Although 127 products may seem sizeable, this does not necessarily translate into a robust antivenom market. Some products have efficacy and safety issues, traditional antivenoms are expensive to produce, and many exist in a loosely regulated environment. Antivenoms are generated against the specific venoms used during animal immunization, with the possibility of also covering venoms of related species (paraspecificity). This method, along with certain products, even predate our system of regulatory approval hence why antivenoms have a different R&D pathway to other biological products. This foremost characteristic has a significant impact on the therapeutic scope of these products since they are effective in some geographical regions only, thus limiting their use in other settings. A growing body of evidence of preclinical efficacy of antivenoms has been built in recent decades (see for example Segura et al., 2010 [21], for the case of Latin America). The latter helps to explain why there is such heterogenous clinical data and highlights the need for robust preclinical assessments until we have the means to run more large-scale clinical trials. Antivenoms save lives and will continue to play a key role in the treatment of SBE, but our data validates a shift towards a dual R&D agenda: focusing both on improving existing antivenoms in the short to medium-term, and on developing more cost-effective and safer, next-generation SBE therapeutics in the longer term. While priorities for improving products already on the market can be framed as short-term goals, their application will shape the R&D ecosystem that future candidates will pass through, for example via improved preclinical standards. Improvements to products now will therefore be a key step in laying the foundation to achieve long-term priorities for next-generation technologies. Table 2 outlines potential next steps that could be prioritised in the SBE R&D community to accelerate progress both in the short and long-term.

**Table 2:** Possible next steps to accelerating SBE R&D.

Activity	Scheme (short / long term)
Establish antivenom paraspecificity and renew efforts for improved preclinical testing	Short-term priority for products to improve coverage and efficacy
Improve manufacturing techniques for traditional antivenoms and invest in existing manufacturers to help them achieve GMP standards	Short-term priority for products to ensure safe and effective antivenoms and to improve products already in the field that do not meet standards
Map out gaps, needs and priorities in the R&D agenda for SBE across different product types, e.g., drugs, biologics, diagnostics, and supportive therapies.	Short-term priority to coordinate and organise the research agenda which may take years to deliver candidates to the market
Develop a prioritisation framework to assess candidates in the pipeline to guide research and funding decisions	
Develop novel diagnostic tests to support diagnosis, epidemiology, and clinical trials	Short-term priority to support design of new products and validation of current products and improve epidemiological and clinical data collection
Align on a chosen governance structure for both researchers and funders to facilitate coordination and harmonisation	Short-term priority to ensure efficient use of resources and knowledge-sharing
Map out category 1 and 2 snakes against antivenoms’ coverage to identify gaps in the market	Short- and long-term priority for products and candidates. Gaps will inform how to redesign current immunisation strategies and prioritise candidates
Update clinical trial protocols including refining clinical endpoints	Short and long-term priority for traditional and next-generation technologies, including combination therapies (e.g., SMT and traditional antivenom)
Conduct fit-for-purpose clinical studies to evaluate safety and efficacy	Short and long-term priority to develop capacity and experience in running clinical trials to generate evidence on SBE products and candidates
Continue to collect data and produce analysis on the state of the research pipeline and market, research funding, cost-effectiveness, and impact of SBE interventions and return-on-investment	Short and long-term priority to ensure the SBE R&D community has the right evidence to make informed decisions and to attract new actors
Develop novel biologics and small molecule therapies to address gaps in traditional antivenoms	Long-term priority with several years of sustained investment
Promote the integration of access perspectives, and coordination with key actors	Long-term priority to ensure that next-generation therapeutics will be accessible once they reach the market

Key ways to improve traditional antivenoms could be achieved through R&D focusing on (1) improving immunisation strategies by selecting venom immunising mixtures that provide wider coverage, (2) introducing new adjuvants to generate a higher immune response, (3) generating new protocols for animal bleeding, (4) improving plasma fractionation protocols, (5) developing robust preclinical assessment of antivenom efficacy and safety within the 3Rs principle within animal testing (i.e., replacement, reduction, refinement) [22], (6) assessing clinical efficacy and safety, and (7) strengthening pharmacovigilance. The combination of sound epidemiological data with proteomics and toxicovenomics analyses provides a robust platform for designing improved venom and toxin mixtures for immunisation. The WHO program of risk-assessment of antivenoms uses a platform of preclinical evaluation of antivenom efficacy that combines antivenomics with neutralisation assays, which enables the assessment of paraspecific antivenom efficacy [23,24]. Prioritising paraspecificity studies using similar designs offers a potential short-term solution for improving existing antivenoms by expanding their coverage, and therefore clinical use.

Furthermore, fit-for-purpose clinical protocols are needed to deliver robust safety and efficacy data to improve confidence in products on the market [25]. As identified in this study and in the literature [10,11], not only is clinical trial data heterogenous but there is also variability in the results and outcomes assessed, meaning the availability of clinical data does not in itself necessarily translate to safe and effective products. Recently, a global core outcome measurement set for snakebite clinical trials was published, an important first step towards standardisation and improving clinical trial feasibility [6]. However, while traditional Phase III studies remain the gold-standard, innovative approaches that could shorten duration and reduce costs of late-stage R&D may be considered in certain strictly defined circumstances such as areas of high incidence of snakebites or seasons involving snakes possessing venoms known to be highly potent and with no available products that are proven effective. In such situations the monitored emergency use of unregistered and investigational interventional (MEURI) products approach may merit consideration.

To achieve these goals, we need to identify and mobilise funders that can invest in all stages of R&D. Although there is funding available for preclinical research, there is a lack of funders that support later-stage research in antivenom manufacture and testing. As an example, the European and Developing Countries Clinical Trials Partnership and African Pharmaceutical Technology Foundation have the right mandates for clinical trials and manufacturing activities but are not involved in the SBE space. A key issue is the need to fund antivenom manufacture in countries with high incidence of SBE. This involves funding for R&D activities, as well as for improvements in infrastructure, equipment, training of staff, and implementation of Good Manufacturing Practice (GMP). While there is room for improvement in the antivenom market, some gaps cannot be addressed by improving efficacy, access, and coverage alone. Other weaknesses of current antivenoms include the fact that they must be administered by trained health workers in health centres, and have limited efficacy to abrogate local tissue damage inflicted by snake venom [17]. There is therefore a need for treatments which can be administered in the field, like heat-stable SMTs that can inhibit necrotising and haemorrhagic toxins, buying time for victims of snakebite and lessening its overall burden [17,26], as well as novel biologics which in the long-term could be cheaper to manufacture, more specific and less immunogenic [27].

Similar to the product market, 197 investigational candidates could easily be (mis)interpreted as a full pipeline. However, the aim of this database was to canvas everything that has been researched or was on the market without making a value-based judgment on the actual therapeutic potential, meaning there are candidates included which are likely to have low therapeutic value or tractability. The purpose was to build a comprehensive baseline of everything in motion, with the intention to then narrow down and prioritise. With this in mind, what constitutes a “real” candidate is a key question. While therapeutic efficacy is essential, a high-potential candidate must also exist within a system where its development can be supported, and where it has a realistic deployment plan in terms of manufacturing and access. Currently, the asymmetry in funding and support for research (early-stage vs late-stage and manufacturing) and production capacity (geographic heterogeneity, e.g., Africa) is hindering progress. There is a pressing need to clarify the R&D agenda to both prioritise research and improve the ecosystem for research-to-market translation. Addressing this requires reflection on the existing R&D ecosystem and overcoming bottlenecks inhibiting progress and coordination.

These major roadblocks are evident in the immature state of the research pipeline. Despite increased investment and focus on novel recombinant antibody research for SBE [8], there has been limited translation of candidates out of basic and early-stage in the last seven years. There are several reasons for this. Despite promising advances [28,29], the first barrier is the diversity of relevant toxins in snake venoms, which represents a challenge in developing broadly neutralising recombinant antibodies. While advancements have been made, they remain largely focused on inhibition of specific toxin subfamilies and are not yet sufficiently developed to be combined and tested in humans.

Although it is promising to see growing interest in the field of recombinant antivenoms, research projects are largely undertaken by academic institutions, reflecting a sector mostly comprised of basic research, with very few academic actors having the capacity or means to translate findings into clinical studies and then manufacture. In addition, there is no clear regulatory pathway for this new type of recombinant antibody therapy for SBE; it is important that discussions on regulatory frameworks for these technologies be developed in parallel with R&D [30]. Lastly, with the cost of development increasing with the complexity of clinical trials and regulatory frameworks, the current lack of diversity of funders to support late-stage research is seriously hindering progress and translation.

On the other hand, repurposed drugs have shown great success in other NTDs as being faster and cheaper to develop. Indeed, methyl-varespladib and unithiol, two repurposed drugs, have emerged as the fastest-moving SBE candidates and are already in clinical trials. However, given the diversity of snake venom toxins, it is likely that SMTs will need to be applied as combination therapies either with traditional antivenoms, novel biologics, or a cocktail of SMTs [28,31]. Combination therapies add an extra layer of complexity to clinical trials but are likely to be necessary to ensure broad acting, safe and effective treatments.

To push biologic and drug candidates through the pipeline, a properly equipped ecosystem, which includes a diverse range of actors with the means and experience to develop and register products, will be critical. The current SBE R&D landscape has a distinct lack of large industry players and multinational companies, or specialised Product Development Partnerships, which historically have been the big movers of NTD products. More coordination is also needed between existing actors, ideally through innovative Public-Private-Partnerships to spur research between partners with different strengths. Looking ahead, the community must be prepared for how a successful recombinant antibody or SMT will be rolled out, at accessible cost, to low-resource or hard-to-reach settings, where most snakebites occur.

Many questions have been raised by this research, including which avenues should be prioritised for SBE R&D, how to identify a promising candidate, how to address the asymmetry in research and production capacity, and what a productive R&D ecosystem could look like (and who should be in it). The study presents a comprehensive baseline but still has limitations, including the possibility of missing candidates and products for several reasons (see S1 Text for full details) and static information current as of September 2022. This calls for further studies using additional sources of information, particularly in countries with high incidence of SBE. Data interoperability will be key for moving forward, harmonising existing data, and generating new evidence on different aspects of the SBE issue to provide a comprehensive view of where different fields intersect. For example, intellectual property (IP) is a topic that requires further investigation. Although this does not seem to be an issue in the case of antivenoms, it may have implications in terms of costs and accessibility in the case of new drugs and recombinant antibodies. This aspect should be explored in future studies, and public health authorities should consider the implications and possibilities of IP in the treatment of SBE [32]. Ongoing research and focused stakeholder collaboration will enable the community to start answering these questions. By mobilising partners, funding and coordination, a serious effort to accelerate the improvement and development of the products and candidates identified in this landscape could be possible. Ideally, we foresee an established governance structure of funders and researchers, and a realistic understanding of the community’s capacities and goals, as well as the needs of public health systems, as kickstarting a new phase in the SBE sector where all actors converge to work towards a common and unified R&D agenda.

## Supporting information

S1 Text Methodology for development of Snakebite Envenoming Medicines Database for therapeutics: investigational candidates and marketed products, 2015–2022). (DOCX)

S2 Text List of included marketed and/or available products. (DOCX)

S3 Text List of included investigational candidates. (DOCX)
