# Supplementary material for: Landscape of toxin-neutralizing therapeutics for snakebite envenoming (2015–2022): Setting the stage for an R&D agenda
Source: PLoS Negl Trop Dis. 2024 Mar 26;18(3):e0012052. doi: 10.1371/journal.pntd.0012052 (PMC10965046; doi:10.1371/journal.pntd.0012052)
Supplement: S1 Text — (DOCX) [file pntd.0012052.s001.docx]

Supplementary material S1.

Methodology for development of Snakebite Envenoming Medicines Database for therapeutics: investigational candidates and marketed products, 2015-2022)

This methodology was adapted from Policy Cures Research’s Accelerating Impact for Mothers (AIM) project on the maternal health medicines pipeline, as described in previous publications.^1-4^ The snakebite-specific methodology has also been detailed in Policy Cures Research white paper “Landscape of Medicines Use & Development for Snakebite Envenoming (2015-2022)”.^5^

Our approach was to create a comprehensive database profiling a) all products registered and/or available for snakebite envenoming (with direct action on toxins) since 2015 (‘products’), and b) all drugs and biologics investigated as potential snakebite therapeutics (with direct action on toxins) since 2015 (‘investigational candidates’). Products and candidates could be applicable for use in any context, including high-income country (HIC) and low- and middle-income country (LMIC) contexts.

For inclusion in the dataset, the products and candidates needed to:

- Be synthetic or natural small molecules (drugs) or immunoglobulin (Ig) (animal plasma/serum-derived or recombinant) or non-immunoglobulin (non-Ig) (animal, natural or recombinant) based biological therapies (biologics), with no restrictions: entries could be entirely new chemical or biological entities (NCEs) or existing/repurposed/label extensions
- Have a direct inhibitory action on snake venom toxins, neutralising venom components to have a therapeutic effect on snakebite envenoming
- Have evidence of research and development (candidates) towards product development, or use (products) at any point since 2015
- Be either investigated for potential clinical use and/or used currently in clinical treatment of snakebite envenoming from WHO medically important category 1 or 2 snakes (or both) only

Specific exclusions were:

- Adjunct and supportive therapies which only modify immune responses and symptoms caused by snake venom toxin but do not have direct action on toxins
- Devices, diagnostics and other non-medicine-related biomedical products with an indication for SBE
- Basic and fundamental research which was not geared towards product development

We undertook a series of partially sequential, partly overlapping, but mutually reinforcing steps to develop a database of product and candidate profiles. These were:

1. identify and validate candidates and products through multiple sources that are, or were, in use or development since 2015;
2. collect information on the products’ and candidate’s preclinical and/or clinical development, and associated data;
3. research additional context around the products and candidates (e.g. immunisation/production strategy, paraspecificity, region of use and/or registration etc.) to build out multi-field entry profiles; and
4. validate and sense-check product and candidate profiles through independent, external reviews by experts in the field.

Data requirements

These four research steps were borne out of an initial data requirement gathering exercise, whereby we agreed to and defined data fields to be captured for each product or candidate (where available and verifiable) (see S1 Table 1 and S1 Table 7 for definitions and examples).

S1 Table 1. Data fields captured for each product or candidate (where available).

| **Product or Candidate profile** |  |
| --- | --- |
| Identifier | Development lifecycle |
| Candidate ID (internally assigned number) | Current R&D stage |
| Candidate name | Highest R&D stage |
| Alternative names | Development status |
| Chemical name | Developers |
| CAS number | Known funders |
| Patent | Preclinical results status |
| Adis Insight ID | Type of preclinical results |
| Adis Insight URL | Preclinical results source(s) |
| Use-case | Inactive development type |
| Disease | Inactive development reason |
| Main product type | Researched in pregnant women or lactating women |
| Sub product type | If marketed, regulatory approval type/level |
| Indication | Evidence tested in clinical trials (Y/N) |
| Investigated for other indications (Y/N) | If yes, does clinical trial evidence pre-date 2015 (Y/N) |
| Other indications | Registration details (products only) |
| Thermostability | Clinical use status |
| Technical profile | Approval status |
| Archetype | Approving authority |
| Target | National Authority Approval status (and date) |
| Route of administration | US FDA approval status (and date) |
| Mechanism of action | EMA approval status (and date) |
| MeSH headings / pharmacological class | Japanese MHLW approval status (and date) |
| Key features and challenges | Other stringent Regulatory authority approval (and date) |
| Recent updates | Stringent Regulatory Authority (SRA) approval (and date) |
| Snake species (product derived from) | WHO pre-qualification (and date) |
| Snake family (product derived from) | Countries where the product is approved |
| Snake species risk category (product derived from) | Region of use |
| Immunizing venom protocol (monospecific/polyspecific) | Approved for use in pregnant or lactating women |
| Region (snake/venom derived from) | FDA pregnancy labelling/pregnancy risk summary |
| Country (snake/venom derived from) | Linked clinical trial data (if applicable) |
| WHO immunising species | CT number |
| WHO paraspecificity species | CT title |
| Ig final product type/preparation | CT description |
| Ig format - animal derived | CT phase |
| Ig format – recombinant | CT status |
| If Ig format - recombinant ‘other', specify | CT last updated |
| Production technique and/or immunization strategy | CT start date |
| Snake species (product tested in) | CT start type (anticipated/actual) |
| Snake species effectiveness (any efficacy data) | CT end date |
| Snake family effectiveness (any efficacy data) | CT end type (anticipated/actual) |
| Snake species risk category effectiveness (any efficacy data) | CT recent updates |
| Direct action on toxins (Y/N) | CT source |
| Target toxin class | CT sponsor |
| Target toxin class | CT collaborator |
| Syndromic profiles | CT locations |

For each field, we developed a definition, data input description, and sample data type classification (for example, numeric, free-text, or defined list, etc.), as well as guidance notes where relevant, to ensure standardised data entry across researchers/enumerators.

**Key scope and data decisions**

As research unfolded and some new end-user requirements for the database were introduced, it was necessary to revisit, stress test, and occasionally make ongoing, minor adjustments or clarifications to the inclusion and exclusion criteria, as well as various database fields. Each change or refinement was made with the aim of maximising standardisation and utility across profiles. In general, and when conflicting options arose, our approach erred on the side of inclusivity, in line with our aim to capture the broadest view possible of the SBE R&D landscape. In line with that aim, we made no judgments based on therapeutic potential, including all molecules and biologics tested for direct action against snake venom toxins (as products or as potential therapeutics), regardless of their effectiveness as available products or feasibility as marketable ones. Modifications and decisions were documented, and include the following:

**Refinements related to inclusions/exclusions**

- Only botanical and natural extracts with isolated compounds and metabolites identified and tested for direct action on toxins were included. Crude botanical extracts (e.g., ethanoic preparations) were excluded, even when tested for direct action on snake venom toxins and neutralisation capacity.
- All levels and types of efficacy data were included, whether *in vitro*, *in vivo*, lethality, toxicity, partial or full. This was a practical decision, as well as one of inclusivity, to enable efficient input of (already) multiple levels of data.
- Novel immunogens to improve immunisation techniques for immunoglobulin-based products were excluded, unless the resulting antibodies or antisera generated were tested for snake venom neutralisation ability (the latter being the candidate entry).
- Discovery and very early research, and research programmes or projects with no specific lead candidate identified but with a clear intention towards product development were included as candidates. This was specifically to ensure the broadest view of the R&D landscape for SBE was captured, especially the nascent stage of most SBE R&D. To avoid duplication, where projects were also able to be validated by unique lead candidates subsequently identified in the literature, the lead candidate was included and the project entry then excluded.
- Multiple potential candidate entries identified in one piece of research – e.g., a series of antibody fragments developed and tested, or multiple plasma-derived antisera with varying venom profiles generated and tested – were entered as unique entries only where it was feasible (i.e., numbers were few enough to not overly distort the landscape) and made sense to do so (i.e., units included different product types or subtypes or had very different profiles). Where not feasible, or where separation didn’t make sense (i.e., where product types were in a series, venom profiles were similar, or where the intention was clearly for one end product such as a polyspecific antivenom), only one entry was created.
- Any candidate or product with evidence of use or development were included, even if they were essentially inactive. For example, FAV-Afrique and Favirept were included – despite their last vials said to have expired around 2016 and both being technically discontinued – based on their use within the project timeframe as well as their future status for re-introduction (i.e., making them active products).
- Products or candidates with action against envenoming from snakes outside of risk categories 1 and 2 but where need and medical importance clearly exists (e.g., sea snakes), were excluded to preserve the focus on snakes with high medical importance.
- Candidates or products with different formulations or different routes of administration were entered as separate entries if the products were substantially different in bioavailability and R&D progress (e.g., varespladib vs methyl-varespladib). Lyophilised and liquid formulations of the same antivenoms were not disaggregated.

**Refinements related to data fields**

- *Archetype:* “Repurposed” products or candidates were any molecules or biologics previously investigated (“repurposed (investigational)”) or marketed (“repurposed (approved)”) for any other condition (this includes approval as dietary supplements or food additives). These two repurposed categories were included to better capture work within the SBE R&D landscape that leverage products that are not yet marketed but where substantial research already exists. “New chemical or biological entities (NCEs)” were products or candidates not already marketed or investigated for any condition (unless an NCE marketed for SBE).
- *Current R&D stage:* SBE marketed products were assigned either “preclinical” or “post-marketing human safety/efficacy studies (without prior clinical studies)”. The latter was created specifically to reflect the different product development and regulatory pathway for antivenom products for SBE compared with products for other neglected diseases. The gold standard (and current minimum requirement) for antivenom approval requires only preclinical venom-induced neutralisation lethality studies *in vivo*. Most clinical studies (if undertaken) are therefore conducted post-marketing without preceding Phase I – Phase III human trials. For this reason, the “post-marketing human safety/efficacy studies (without prior clinical studies)” label is not synonymous with Phase IV, given no prior phase development progress is required to progress to marketing authorisation. SBE investigational candidates were assigned labels that follow traditional R&D pathways “discovery & preclinical”, and “Phase I, II or III” (even though it is unlikely that – at least for novel traditional animal-plasma derived antivenoms – there will be a requirement for more than minimum preclinical data for approval in the foreseeable future).
- *Valency/specificity:* Following expert advice, we defined product ‘valency’ in its strictest (traditional) sense, as a reflection of the number of species of snakes used in the venom immunising protocol (for immunoglobulin biologics), i.e., single snake species source as monospecific and multi-snake sources as polyspecific. We renamed this field as “immunising venom protocol” to improve clarity. We included separate sections for “snake species (product tested in)” and “snake species effectiveness (any efficacy data)” to capture entries’ snake specificity/paraspecificity.
- *WHO immunising species/WHO paraspecificity species:* We found discrepancies between sources related to marketed antivenoms’ immunising snake species and listed paraspecificity, including between manufacturers’ websites and the WHO. For comprehensiveness and to avoid the need to judge accuracy, we included both, adding two additional fields to capture information from the WHO source.
- *Region of use/registration:* For a more accurate view of the spread of available SBE products, we added a field for “region of use”, alongside “region of registration”. The former allowed us to better reflect the fact that sometimes antivenoms are produced by an institute or manufacturer well outside of the region of intended use, meaning these are not synonymous. However, data in general, including on regulatory approval status and location, was difficult to find and so we relied heavily on information from the WHO. It was challenging to reflect this in the selected fields, so where possible we added notes in the “key features and challenges” section of the product profiles.

Methods and sources

Step 1: Initial candidate identification

Various sources were utilised to uncover and identify a total of 127 marketed or available products and 196 investigational candidates.

(a) We searched Adis Insight^6^ – a leading drug development database – to retrieve a comprehensive and up-to-date output of relevant drugs and biologics under investigation for snakebite envenoming. The platform returns detailed information on drugs, candidate deals, clinical trials, safety, patents, and other historical information useful for building candidate profiles. Information is full via subscription (our approach) or limited via open source. We searched utilising Adis Insight’s inbuilt ‘by indication’ function, which classifies medicines using a standardised list of indications. Accordingly, free-text searches by indication are not possible. We therefore used search terms that were the most relevant, available indications in the database for snakebite envenoming. These were: “snake venom poisoning”, “snake bite poisoning”, “snake bites” and “poisoning by venomous snakes”^^[[1]](#footnote-1)^^. We also searched using the ‘by drug class’ search function (also not free-text), with the pre-formed relevant search terms “snake venoms”, “polyvalent snake antitoxins”, “polyvalent snake antivenins”, and “polyvalent snake antivenom”. Lastly, we searched using the free-text option ‘All Text’ with the search term “snake”.

“Drug” and “trial” outputs were retrieved, triangulated and de-duplicated, and unique products, candidates and associated data formatted, extrapolated, and transposed into our database. Adis Insight search results were retrieved in March 2022 (see S1 Table 2).

S1 Table 2. Number of products and/or candidates retrieved from Adis Insight

|  | **Products** | **Candidates** |
| --- | --- | --- |
| Number of unique returns from search terms via Adis Insight | 43 | |
| Number of unique entries identified via Adis Insight as in scope | 4 | 4 |

b) We searched the WHO Snakebite Information and Data Platform^7^, specifically the table on Antivenom and Manufacturers. The database is a comprehensive overview of risk category 1 and 2 snakes, their associated geographical distribution and potential health impact and disease burden. It also lists marketed and available antivenom products with known use and/or use efficacy against specific snake species, as well as information on registration and WHO assessment status. The data platform is ‘snake focused’ as opposed to ‘antivenom focused’, and as such data retrieved needed to be de-duplicated and re-oriented to identify all available marketed products. Results were retrieved in March 2022 (see S1 Table 3).

S1 Table 3. Number of products and/or candidates from the WHO Snakebite Information and Data Platform

|  | **Products** | **Candidates** |
| --- | --- | --- |
| Number of unique antivenoms retrieved via the WHO SBE data platform | 120 | 0 |
| Number of unique antivenoms identified via WHO SBE data platform as in scope | 120 | 0 |
| Number of additional profiles identified via WHO SBE data platform not already identified via Adis Insight | 116 | 0 |

c) We exported datasets from the WHO International Clinical Trials Registry Platform (ICTRP)^8^ the most comprehensive list of global clinical trials available. The following search terms were used to retrieve datasets – which were then merged and deduplicated into one – related to SBE in March 2022: “snake”; ‘snakebite”; “envenoming”; “snakebite envenoming”; “antivenom”; and “antiserum”. Clinical trials were scoped for relevance, which we defined as an investigation of one or more drugs or biologics with a primary and/or secondary outcome measure matching treatment of snakebite envenoming, and where the mechanism of action was direct action on snake venom toxin (i.e., not adjunct or supportive therapies). This data search served a dual function of uncovering additional products and/or candidates for inclusion that had not yet been identified (see S1 Table 4), as well as capturing and linking clinical trial data to candidates and products marked for inclusion in the database (see Step 2 below). We performed a similar search of clinicaltrials.gov, but since this information is already contained within ICTRP, no additional candidates or products were identified outside of the figures quoted below.

S1 Table 4. Number of candidates and products from ICTRP

|  | **Products** | **Candidates** |
| --- | --- | --- |
| Number of unique clinical trials returned via ICTRP | 103 | |
| Number of unique clinical trials identified as in scope | 32 | |
| Number of unique profiles identified via in scope clinical trials | 19 | 4 |
| Number of additional profiles identified via ICTRP not already identified via Adis and WHO SBE data platform | 1 | 3 |

(d) We searched PubMed^9^ for relevant literature to validate already identified products and candidate and uncover new ones for inclusion. We anticipated this would include several entries – particularly candidates in discovery and preclinical development – and thus considered search terms that would return information on novel or innovative R&D. We searched using the same search terms as those in our other searches related to the condition/disease (“snakebite”, “envenoming”), combined with additional terms related to indication (“treatment”, “therapy”), product type (“antivenom”, “drug”, “biologic”), and innovation (“innovation”, “discovery”, “preclinical”, “novel”). Using these terms, we performed the following search combinations (using all available terms and combinations):

- “*condition” (i.e., “snakebite”)*
- *“condition” + “indication” (e.g., “snakebite” + “treatment”)*
- *“condition” + “product type” (e.g., “snakebite” + “antivenom”)*
- *“condition” + “indication” + “innovation” (e.g., “snakebite” + “treatment” + “novel”)*
- *“condition” + “product type” + “innovation” (e.g., “snakebite” + “antivenom” + “novel”)*

PubMed searches were conducted in March 2022. All searches were combined and deduplicated, and returned paper titles and abstracts reviewed for relevance. Relevant publications were reviewed in full. Unique and in-scope candidates were added to the database, or additional data on existing candidates already entered in the database was captured (see S1 Table 5).

S1 Table 5. Number of publications (total/relevant*) retrieved, and candidates and products (additional) identified from PubMed

|  | **Products** | **Candidates** |
| --- | --- | --- |
| Number of publications (total/relevant*) retrieved | 1109/454 | |
| Number of additional candidates identified via PubMed not already identified elsewhere | 7 | 167 |

^* Relevant papers are those in which we identified additional or validated existing candidates or products^

(e) We searched the grant databases of three of the largest global funders of medicines development to validate existing and find new products and candidates: the United States National Institutes of Health (US NIH)’s RePORTER^10^; the European Union/Commission’s CORDIS^11^; and the Bill & Melinda Gates Foundation grants database (data supplied from the Foundation); as well as those of two major global funders of SBE therapeutics R&D with available online data: Wellcome’s grant funding^12^ and the US Department of Defense’s via USASpending.gov^13^. This served to validate existing, and find new, candidates and products (particularly those in preclinical/discovery stage). For all databases, we searched using the same search terms as described above, in various combinations. For RePORTER, we retrieved all grants dating from 2015 to present. For CORDIS, we retrieved the Horizon 2014–2020 dataset. For the Gates Foundation, we searched datasets ranging from 2014–2019 inclusive (dates supplied and available for review). For Wellcome, we retrieved all grants available for review (from 2005 to present), and for the US DOD, we retrieved grants from 2015 to present. We also searched our own internal G-FINDER R&D funding database for relevant projects. G-FINDER began systematically tracking SBE R&D funding in 2018 and at the time of this project had data available to 2020. We identified other funders and developers through this review and searched their websites to fill gaps.

All datasets were retrieved and scoped for relevance in March 2022. Profiles were created for new candidates or information added to existing candidates, as appropriate (see S1 Table 6).

S1 Table 6. Number of grants, candidates and products retrieved from donor databases/sources

|  | **Products** | **Candidates** |
| --- | --- | --- |
| **RePORTER** | | |
| Number of grants returned via RePORTER | 84 | |
| Number of grants identified as in-scope | 14 | |
| Number of unique candidates identified via in-scope grants | 1 | 5 |
| Number of additional candidates identified via in-scope grants not already identified elsewhere | 0 | 4 |
| **CORDIS** | | |
| Number of grants returned via CORDIS | 22 | |
| Number of grants identified as in-scope | 2 | |
| Number of unique candidates identified via in-scope grants | 0 | 1 |
| Number of additional candidates identified via in-scope grants not already identified elsewhere | 0 | 1 |
| **BMGF** | | |
| Number of grants returned via BMGF | 1 | |
| Number of grants identified as in-scope | 0 | |
| Number of unique candidates identified via in-scope grants | 0 | 0 |
| Number of additional candidates identified via in-scope grants not already identified elsewhere | 0 | 0 |
| **WELLCOME** | | |
| Number of grants returned via WELLCOME | 29 | |
| Number of grants identified as in-scope | 13 | |
| Number of unique candidates identified via in-scope grants | 1 | 12 |
| Number of additional candidates identified via in-scope grants not already identified elsewhere | 0 | 10 |
| **US DOD** | | |
| Number of grants returned via US DOD | 34 | |
| Number of grants identified as in-scope | 4 | |
| Number of unique candidates identified via in-scope grants | 1 | 1 |
| Number of additional candidates identified via in-scope grants not already identified elsewhere | 0 | 0 |
| **G-FINDER** | | |
| Number of grants sourced from G-FINDER | 95 | |
| Number of grants identified as in-scope | 70 | |
| Number of unique candidates identified via in-scope grants | 0 | 9 |
| Number of additional candidates identified via in-scope grants not already identified elsewhere | 0 | 6 |

Step 2: Linking preclinical and clinical development data

For candidates in clinical development, we collected relevant clinical trial data through a few sources. Primary candidate and product identification through Adis Insight (Step 1) also provided linked clinical trials. These were scoped for relevance, and manually uploaded to the clinical trial entries in our database. Next, we datamined the datasets retrieved from the WHO International Clinical Trials Registry Platform (ICTRP) as described above. We scoped every clinical trial entry in the dataset, relevant trials were marked for inclusion and assigned to a candidate or product (or multiple if more than one was being investigated). Given the size (in the thousands), this data was then uploaded to the clinical trial entries in our database using a coded, automated upload. We cross-checked ICTRP clinical trials with those from Adis Insight and clinicaltrials.gov to rule out duplicates. Clinical trial data review was performed between March and April 2022. For candidates in preclinical development, results were sourced through PubMed searches between March and April 2022 (see Step 3).

Step 3: Completing candidate and product profiles

Much of the candidate information needed to complete candidate profiles was provided through Steps 1 and 2. In addition, we used academic literature search engines/tools, manufacturer websites and grant descriptions to source greater detail and context for the candidates and products identified in Steps 1 and 2. Primarily, we searched PubMed using the candidate and product name(s), and reviewed relevant literature retrieved (including that already sourced in Step 1) to verify and cross-reference information as needed. This type of information helped us to understand the development status/activity of candidates and products, as well as provided deeper information on those in preclinical development. Additional information was searched for via relevant regulatory websites, such as the US Food and Drug Administration (FDA)^14^ and European Medicines Agency (EMA)^15^ as appropriate, as well as a number of reliable online sources, including DRUGBANK Online^16^, PubChem^17^, the US National Library of Medicine’s Medical Subject Headings (MeSH) portal^18^, and other websites as needed. Technical information on products was primarily sourced from developer websites and where possible, directly from antivenom product pamphlets/inserts.

Additional candidate profile information was conducted between March and June 2022, concurrently with the steps outlined above.

Step 4: External validation and sense checking

Following database completion, a series of internal and external, independent reviews were undertaken to clean and validate the data. Internally, each candidate was reviewed for content, consistency, and logic by a minimum of two individuals. Data cross-checking and cleaning was conducted in a rigorous, sequential manner. Some steps served to clean and standardise the data, while others were intended to identify content or subject matter error. Illustrative content checks included, for example, reviewing archetype against highest R&D stage (i.e. all “repurposed” candidates needed logically to have a highest R&D stage as “marketed”), or reviewing snake venom sources against country and region of origin and specificity (i.e., monospecific antivenom logically listing only one snake species, one country, and one region as venom source).

An external review was also undertaken. We sought independent, specialist input from members of the project’s expert advisory group: Ian Cameron (MicroPharm), Nicholas Casewell (Liverpool School of Tropical Medicine), José María Gutiérrez (Instituto Clodomiro Picado, University of Costa Rica), Andreas Hougaard Laustsen-Kiel (Technical University of Denmark), Matthew Lewin (Ophirex / California Academy of Sciences), Julien Potet (MSF Access) and Devin Sok (IAVI).

The entire database was reviewed to validate or identify known missing products and candidates; review the essential, standard labels; The entire database was reviewed to validate candidates or identify known missing candidates or products; review the essential, standard labels for the candidates; provide additional industry information to fill gaps as appropriate; and for each, recommend corrections, improvements, or additional details. External review was undertaken between June and July 2022.

Materials and platforms

Our data was entered and stored in a bespoke database built in Microsoft CRM Dynamics. Data was transposed (when needed) to Microsoft Excel and Word. Our analyses were performed using Microsoft Excel.

Limitations

Our aim was to identify all drugs and biologics in use or in development for SBE since 2015, which we approached by utilising the comprehensive, multi-pronged search strategy described above. However, due to the nuanced nature of the SBE medicines R&D landscape and inherent biases and weaknesses in available information, the database has some limitations.

Firstly, due to the proprietary nature of (and lack of publicly available information on) many, particularly preclinical, candidate investigations, we anticipate the data may have gaps with respect to the full body of research. We also acknowledge that nearly all the data sources used rely on self-reporting by investigators, which have their own inherent limitations, including potential for reporting biases arising from changes in adherence and utilisation over time, as well as publication biases towards positive results. Other data may have been overlooked due to English-language bias, especially for Central and South American products where much of the available information was in Spanish and Portuguese. Lastly, information overall – particularly for available products – was often extremely hard to obtain, sometimes dictating that we rely heavily on secondary sources to fill gaps.

Secondly, our deliberate decision to be as inclusive as possible means also that within the database we are not always comparing like with like. Examples include: projects and programmes of work versus a clear lead drug candidate; alternative animal models giving rise to genuine novel biologics (e.g., chicken antibody and antibody fragment candidates) versus alternative animal models as precursor research to more traditional approaches (e.g. some rabbit antisera candidates, also acknowledging some publish findings with this type of research and others don’t); and single molecules or isolates under investigation versus combined related series of molecules or biologics. Despite these differences, this approach has enabled us to present the most comprehensive database of molecules, biologics and streams of work with therapeutic potential possible, fulfilling our intention to capture what is going on in the SBE R&D landscape in the broadest sense.

Thirdly, direct action on snake venom toxins *sensu stricto* was a major inclusion criterion for this undertaking. This meant, however, that some R&D with alternative mechanisms of action – but similar outcomes and therapeutic potential – were necessarily omitted. This includes, as an example, acetylcholine esterase inhibitors like neostigmine, which offer potential anti-3FTx (three finger toxin) capabilities by altering internal physiological processes (increasing concentration of acetylcholine at the synaptic cleft) as opposed to acting directly on the toxin itself. Other adjunct and supportive therapies that have the potential for profound corrections in the physiologic and syndromic effects of snake venom toxins were also excluded on this basis. Candidates like this could and should be investigated and dealt with elsewhere to broaden the view of the R&D landscape for next generation SBE therapeutics.

The Advisory Committee for this project was composed of representatives from different sectors (research institutions, public antivenom manufacturers, private companies) who are knowledgeable in the field of antivenoms and snake venom inhibitors and are aware of, or involved in, the developments being carried out in several geographical settings. Despite this, it is key to note that the majority of the members of the Advisory Committee belong to developed countries with low incidences of snakebites.

Lastly, readers should also note the data is up to date – and analyses drawn from the status of products and candidates – as of mid-2022.

References

1. McDougall ARA, Goldstein M, Tuttle A, Ammerdorffer A, Rushwan S, Hastie R, Gülmezoglu AM, Vogel JP. Innovations in the prevention and treatment of postpartum hemorrhage: Analysis of a novel medicines development pipeline database. Int J Gynaecol Obstet. 2022 Jun;158
2. McDougall ARA, Hastie R, Goldstein M, Tuttle A, Tong S, Ammerdorffer A, et al. Systematic evaluation of the pre-eclampsia drugs, dietary supplements and biologicals pipeline using target product profiles. BMC Med. 2022;20(1):393.
3. Lim S, McDougall ARA, Goldstein M, Tuttle A, Hastie R, Tong S, et al. Analysis of a maternal health medicines pipeline database 2000–2021: new candidates for the prevention and treatment of fetal growth restriction. BJOG. 2023;130(6):653–63
4. McDougall ARA, Hastie R, Goldstein M, Tuttle A, Ammerdorffer A, Gülmezoglu AM et al. New medicines for spontaneous preterm birth prevention and preterm labour management: landscape analysis of the medicine development pipeline. BMC Pregnancy and Childbirth. 2023;23:525
5. Policy Cures Research. Landscape of Medicines Use & Development for Snakebite Envenoming (2015-2022), 2022 Sept
6. SpringER Nature Switzerland AG. Adis Insight: <https://adisinsight.springer.com/>
7. World Health Organization. Snakebite Information and Data Platform: <https://snbdatainfo.who.int/>
8. World Health Organization. International Clinical Trials Registry Platform: <https://trialsearch.who.int/>
9. National Library of Medicine. PubMed: <https://pubmed.ncbi.nlm.nih.gov/>
10. US NIH. RePORTER: <https://reporter.nih.gov/>
11. European Commission. CORDIS. <https://cordis.europa.eu/>
12. Wellcome. Funded people and projects: <https://wellcome.org/grant-funding/funded-people-and-projects>
13. US Government. USAspending.gov: <https://www.usaspending.gov/>
14. US Government. US Food and Drug Administration: <https://www.fda.gov/home>
15. European Union: European Medicines Agency: <https://www.ema.europa.eu/en>
16. DrugBank. DrugBank Online: <https://go.drugbank.com/>
17. National Library of Medicine. PubChem: <https://pubchem.ncbi.nlm.nih.gov/>
18. National Library of Medicine. Medical Subject Headings (MeSH): <https://meshb.nlm.nih.gov/>

**S1 Table 7. Data fields captured for each product or candidate – definitions and examples**

| **Data field** | **Definition** | **Data input type** | **Data example** | **Additional notes & further definitions** |
| --- | --- | --- | --- | --- |
| **IDENTIFIER** |  |  |  |  |
| Candidate ID | Three-digit unique identifier of candidate, assigned internally by PCR. | Numeric | e.g. 101 | N/A |
| Candidate name | The current candidate name, or if not active, the name it had at the time it was last active. International non-proprietary name if available. | Free text | e.g. Varespladib | N/A |
| Alternative/previous candidate names | Any other previous names the candidate has had or been referred to as. Written as list separated with semi-colon. | Free text | e.g. A-001; A-002; LY-315920; LY-333013; Methyl-varespladib; S-3013; S-5920; Varespladib methyl | N/A |
| Chemical name (if appropriate) | IUPAC chemical name in words | Free text | e.g. (2R,3S)-N4-Hydroxy-2-isobutyl-N1-[(2S)-1-(methylamino)-1-oxo-3-phenyl-2-propanyl]-3-[(2-thienylsulfanyl)methyl]succinamide (for batimastat) | Can be found in Adis, PubChem or Drugbank etc. |
| CAS number (if appropriate) | Candidate's CAS number | Free text | e.g. 130370-60-4 (for batimastat) | Can be found in Adis, PubChem or Drugbank etc. |
| Patent (where available) | Patent number or code as per patent database. | Free text | e.g. 8 048 414 (for Crotalidae polyvalent immune Fab (ovine)) | Can be found in Adis, PubChem or Drugbank etc. |
| ADIS ID | Candidate's AdisInsight ID number | Free text | e.g. 800010197 | Internal notes. |
| Adis URL | Hyperlink to the candidate's AdisInsight page | Hyperlink | <https://adisinsight.springer.com/drugs/800010197> | Internal notes. |
| **USE CASE** |  |  |  |  |
| Disease | Formal identification as snakebite | Drop-down (list) | e.g. snakebite | N/A |
| Main product type | Whether the candidate is a drug or biologic |  | Biologic Drug | N/A |
| Sub-product type | What sub-type of drugs and biologics the candidates falls within. | Drop-down (list) | e.g.  **Biologic** -immunoglobulin products - animal plasma/serum derived -immunoglobulin products – recombinant -non-immunoglobulin products - (animal/naturally derived; recombinant) -other  **Drugs -** therapeutic - synthetic - therapeutic – natural/botanical | Biologic immunoglobulin products that are animal plasma or serum derived refer to traditional 'antivenoms', whether derived from venom -dependent or venom-independent immunisation pathways.  Biologic immunoglobulin product that are recombinant include human, camelid, chimeric antibodies etc.   Biologic non-immunoglobulin products include whole or partial proteins, peptides, aptamers etc. This category includes **all types,** whether recombinant, synthetically synthesised, or pure animal derived proteins (e.g. enzymes from opossums or human mast cells).  The biologic 'other' category refers to anything that doesn't fit the other categories neatly. For example, the possibility exists for immunoglobulins to be generated in cattle transfected with recombinant human immunoglobulins. These would be animal-derived but human in nature, for example.  Drug small molecule therapies refer to most chemically synthesised small molecules, whether toxin or non-toxin neutralising, and if the former, whether antagonists or inhibitors. The drug 'other' category refers to botanical extracts but ONLY with identified active compounds, properly dosed, controlled and rigorously tested. Crude extracts and traditional medicines that don't fit this criterion are out of scope. |
| Indication | Within SBE, the sub-category of focus and action of the candidate. | Free text | e.g. to prevent SBE victim's reaction to venom and improve efficacy of antivenoms which are not perfectly matched to the snake's venom as an adjunct therapy. | N/A |
| Investigated for other conditions | If the candidate or products has been investigated for other non-SBE conditions | Drop-down (list) | Yes  No | N/A |
| Other conditions | List of other conditions investigated for, if appropriate | Free text | e.g., Acute coronary syndromes | N/A |
| Thermostability | Degrees celsius needed for storage and transportation | Free text | e.g. 25 degrees celsius | N/A |
| **TECHNICAL PROFILE** |  |  |  |  |
| Archetype | What sub-category of product the candidate represents, specifically whether it is repurposed or a new chemical entity. | Drop-down (list) | e.g.,  New chemical or biological entity Repurposed | Repurposed candidates are any candidate previously or currently marketed for any other condition. 'New chemical or biological entities (NCEs)' are candidates not already marketed for any condition, unless it is an NCE marketed for SBE. |
| Target (general) | Pharmacological target the candidate acts on. | Free text | e.g. for Crotalidae polyvalent immune Fab (ovine): snake venom | General pharmacological targets. If unknown, write 'unknown'. |
| Route of administration | How the medicine is administered or taken. If unknown, as is likely with some preclinical candidates, write 'unknown'. | Drop-down (list) | Intravenous Intramuscular Subcutaneous Intradermal Oral Inhaled Nasal Sublingual Other | If unknown, as is likely with some preclinical candidates, write 'unknown'. Likely to be concentrated on IV, IM and PO, but array of options included. |
| Mode of action | How the medicine works. Description needed, rather than re-wording of target. | Free text | e.g. Varespladib is an inhibitor of the IIa, V, and X isoforms of secretory phospholipase A2 (sPLA2). The molecule acts as an anti-inflammatory agent by disrupting the first step of the arachidonic acid pathway of inflammation. | If unknown, write 'unknown' |
| MeSH headings / Pharmacological Class (if applicable) | The candidate's related NIH Medical Subject Headings, listed under the “Pharm Action” , and/or the candidate's WHO ATC 4th level classification or FDC EPC classification. If not available, other keywords to describe the pharmacological or therapeutic uses for the candidate. | Free text | e.g. for Crotalidae polyvalent immune Fab (ovine): Antivenins; Fab fragments; Polyvalent snake antivenins; Immune sera (J06A) e.g. for batimastat/marimastat: Metalloprotease inhibitors; Antimetastatics; Angiogenesis inhibitors e.g. for Varespladib: Phospholipase A2 inhibitors; Acetic acids; Anti-inflammatories; Antirheumatics; Indoles | The MIH MeSh website is (https://meshb.nlm.nih.gov/search). Other useful keywords that describe the pharmacological or therapeutic uses for the candidate can be found through other websites or literature. |
| Key features/challenges | Concise description of the candidate information, in a prescribed format. | Free text | e.g. Antivenom CroFab® is a purified sheep-derived Crotalid rattlesnake antivenom developed by BTG Specialty Pharmaceuticals (a subsidiary of SERB Pharmaceuticals). Conventional antivenom is extracted from the blood of horses that have been injected with venom whereas CroFab® consists of just the purified active portions of the antibodies produced by sheep in response to snake venom. By eliminating the large numbers of nonspecific proteins present in conventional antivenom, the dose can be reduced (to about 20% of that required for the conventional antivenom) and the incidence of adverse events is significantly reduced (from 100% to 10%) .  CroFab® neutralises the venom of crotalids which includes rattlesnakes, copperheads and cottonmouths. It is also effective against diamondback snakes.  CroFab® (formerly CroTAb®) has been launched in the US. The product has been approved by the US FDA for mild or moderate envenomation from North American Crotalid snakes in the USA, where it has an orphan drug status. The product is intended for IV administration.  The primary endpoint of the prospective study was met. The double-blind, placebo-controlled, prospective trial compared CroFab® versus placebo for the treatment of copperhead snake venom poisoning in patients following envenomation. The study compared the scores on the Patient Specific Functional Scale (PSFS) in 74 patients, which were randomised to CroFab® (45 patients) and placebo (29 patients) groups. Patients treated early with CroFab® were fully recovered at day 14. | Descriptions should include general information on the candidates and how it works; any relevant history of its development; and any positive or negatives/challenges of the drug (e.g., side effects), as appropriate. References should be included as URLs in brackets). |
| Recent updates | Concise description of latest update/s - if appropriate. 'Recent' is defined as the last three years (since 2019). | Free text | e.g. May 2021: Moved from in vitro to animal models.' OR e.g. April 2020: Phase II trials in the UK and Australia were discontinued." | Adis Insight already provides information on key recent update (if listed there). |
| Specificity Snake species (product derived from) | The specific snake species (or multiple species)’s from which venom has been used in the immunization protocol and product developed. | Free text | e.g. Boomslang (*Dispholidus typus*); African bush viper (*Atheris squamigera*) | To be included only if this info is available. Write common name followed by scientific name in brackets. If more than one snake species, separate with semi-colon. |
| Specificity snake family (product derived from) | The snake family/ies or sub-family/ies from which venom has been used in the immunization protocol and product developed. | Drop-down (list) - **multiple selections possible** | Viperidae Elapidae Lamprophiidae  Colubridae | Thus, this snake family classification is reflective of the snake family origin of the venom used in production, not the broad scope of efficacy of the antivenom across that family. |
| Snake species risk category (product derived from) | WHO Category 1 or Category 2 species of medical importance, from which venom has been used in the immunization protocol and product developed. | Drop-down (list) | Category 1 (Highest medical importance) Category 2 (Secondary medical importance) Both Category 1 and 2 N/A Unknown | If product includes venom from category 1 and 2 snakes, select 'Both Category 1 and 2). |
| Immunizing venom protocol | Whether the product has been developed using one or more than one type of snake venom, and as such whether it has action against one or more than one snake venom type. (Most relevant to biologics) | Drop-down (list) | e.g.  Polyspecific (broad spectrum, multi-snake venom specificity) Monospecific (single snake venom specificity) | Polyspecific and monospecific refer to are common descriptors, and strictly speaking refer to immunization approaches for antivenom: a 'monospecific antivenom' is an antivenom generated by immunizing an animal with the venom of one snake species, whereas 'polyspecific antivenom' is generated by immunizing animals with venoms from more than one species. By extension however, this is also what the product is effective against (one type of venom vs multiple venom types). (As SMTs are toxin specific, which can be found across venoms, this is largely relevant to biologics).   (NB Syndromic descriptors as emerging antivenoms types (as specified in the WHO TPP) are captured secondarily via the syndromic profile/class below). |
| Region (snake/venom derived from) | Geographical origin of the venom/s used in production, not the broad geographical scope of efficacy of the antivenom. However by extension, the product will have efficacy within, but not across the entirety - of the specified region. | Drop down (list) - **multiple selections possible** | e.g. suggested dropdowns: North Africa East Africa West Africa Central Africa Southern Africa South Asia South East Asia Central Asia East Asia Middle East North America Central American South America Australo-Papua (including Pacific Islands) Central Europe Western Europe Eastern Europe Multiple regions | Multiple regions as a category is most useful to SMTs, which act against toxins present in snakes across regions and continents. For other products, one or more regions can be selected. |
| Country (snake/venom derived from) | Country of origin of the venom/s used in production, not the geographical efficacy of the antivenom. However by extension, the product will have efficacy for some snakes within, but not for the entirety, of snakes within the specified country. | Free text | e.g. Botwana; South Africa | If multiple countries, separate by semi-colons. |
| WHO Immunizing species | WHO listed the snake species from which venom has been used in the immunization protocol and product developed. | Free text | e.g. Boomslang (*Dispholidus typus*); African bush viper (*Atheris squamigera*) | Source is WHO Snakebite Information and Data portal |
| WHO paraspecificity species | WHO listed snakes against which the product has efficacy | Free text | e.g. Boomslang (*Dispholidus typus*); African bush viper (*Atheris squamigera*) | Source is WHO Snakebite Information and Data portal |
| Immunoglobulin final product type/preparation (if applicable) | If an IgG product, whether the final product is a liquid final product or lyophilized final product | Drop-down (list) | Liquid final product Lyophized final product | Only relevant to Immunoglobulin products. |
| Immunoglobulin format - animal derived (if applicable) | If it is an animal derived immunoglobulin product, what format (type and size). | Drop-down (list) | e.g.  -Intact IgG immunoglobulin molecule (whole)  -F(ab')2 immunoglobulin molecule fragments -Fab immunoglobulin molecule fragments  -unknown | Only relevant to Immunoglobulin products. Options to be linked to product sub-type field to only show relevant fields depending on previous selection. |
| Immunoglobulin format - recombinant (if applicable) | If it is a recombinant immunoglobulin product, what format (type and size). | Drop-down (list) | e.g. IgG Fab Single-chain variable fragments (scFv) VHH VHH-Fc VH-oligomers  other  unknown | Only relevant to Immunoglobulin products. Options to be linked to product sub-type field to only show relevant fields depending on previous selection. |
| Immunoglobulin format – other (if applicable) | If Ig type not covered by animal-derived or recombinant options | Free text | e.g. IgG plus VHH (combined) | Only relevant to Immunoglobulin products. To cover the spectrum of possible immunoglobulin formats, this field is free text. |
| Production technique and/or immunization strategy | What type of technology is used to produce the medicine. | Free-text | e.g. potential examples of free text: - Immunization of animals for antivenom production using venom (sub-lethal dose direct) - Immunization of animals for antivenom production using toxin-encoded DNA plasmids - Immunization of animals for antivenom production using synthetically produced short peptides/epitopes  - Immunization of animals for antivenom production using recombinantly produced full length toxins - Immunization of animals for antivenom production using mimotopes with neutralizing capacity against mAbs - Development of recombinant human antibodies using phage display technology - Traditional lab-based chemical synthesis of small molecules - Ethanol extraction of botanical active compounds etc..  -synthetic chemistry | This will allow additional information about the production approach for the specific candidate, including distinguishing between venom-dependent and venom-independent immunization techniques for antivenom production, as well as various approaches to recombinant production, and small molecule lab synthesis etc. This is free text to allow for an array of options (consolidation and wrapping up into broader fields is possible later). |
| Snake species (product tested in) | The specific snake species (or multiple species) that the product is tested in | Free-text | e.g. Boomslang (*Dispholidus typus*); African bush viper (*Atheris squamigera*) | Any evidence of species against which the product has been tested, regardless of outcome. |
| Snake species effectiveness (any efficacy data) | The specific snake species (or multiple species) that the product has demonstrated efficacy against | Free-text | e.g. Boomslang (*Dispholidus typus*) | Any evidence of effectiveness/ neutralisation capacity , *in vitro* or *in vivo* |
| Snake family effectiveness (any efficacy data) | The snake family (or multiple families) that the product has demonstrated efficacy against | Free-text | e.g. Colubridae | Any evidence of effectiveness/ neutralisation capacity , *in vitro* or *in* *vivo* |
| Snake species risk category effectiveness (any efficacy data) | WHO Category 1 or Category 2 species of medical importance, for which the product has evidence of efficacy against | Drop-down (list) | Category 1 (Highest medical importance) Category 2 (Secondary medical importance) Both Category 1 and 2 N/A Unknown | If product includes venom from category 1 and 2 snakes, select 'Both Category 1 and 2). |
| Direct action on toxins? | Whether the product works directly on snake toxins, or not (i.e. has only syndromic anti-inflammatory action). | Drop-down (list) | e.g. Yes; No; Unknown | N/A |
| Target toxin class (if applicable) | Snake toxin class target the candidate acts on. | Drop down (list) | High toxicity toxins Low toxicity toxins Both | First filter to distinguish products targeting the big four (plus dendrotoxins and sarafotoxins), from all the rest (low toxicity). This will make cutting the data easier, especially since greatest interest is in high toxicity toxins from a public health perspective. It is less likely that there will be products with cross-reactivity across high and low toxins, but the 'both' category is added in case. |
| Specific target toxin class (if applicable) | Snake toxin class target the candidate acts on. | Drop down (list) - **multiple selections possible** | e.g. suggested dropdowns: High toxicity - SVMPs High toxicity - PLA2s High toxicity - 3FTxs High toxicity - SVSPs High toxicity - dendrotoxins High toxicity - sarafotoxins Low toxicity - LAAO Low toxicity - SNACLECs Low toxicity - CRISPs Low toxicity - disintegrins Low toxicity - BPPs Low toxicity - hyaluronidase Low toxicity - 5'nucleotidease Low toxicity - NGF Low toxicity - phosphodiesterase Low toxicity - natriuretic peptide Low toxicity - acetylcholinesterase | All snake toxins listed for comprehensiveness, but multiple selections possible. Options to be linked to above field to only show high toxicity toxins, low toxicity toxins or both, as option depending on previous selection. |
| Syndromic profiles | Classifications assigned to candidates based on target/indication/use-case. | Drop-down (list) **multiple selections possible** | e.g., neurotoxic (paralysis) haemorrhagic (bleeding) cytotoxic (tissue damage) procoagulant (blood clotting) N/A Unknown | Only recorded if specified (not inferred). |
| **DEVELOPMENT LIFECYLE** |  |  |  |  |
| Current R&D stage (for SBE) | The current R&D stage of the candidate or product for SBE. If not active, the R&D stage the candidate was at for the condition and indication specified above when it stopped being active. Written exactly as presented. | Free text | e.g., ‘Discovery’, ‘Preclinical, 'Phase II', 'Phase IIb/III', 'Phase III'; ' Post-marketing human safety/efficacy studies (without prior clinical studies)' | To allocate a single, appropriate R&D stage to each candidate, review linked clinical trials against accepted definitions of each R&D stage (preclinical through to Post-marketing studies), and assign a phase based on trial descriptions. |
| Highest R&D stage (for any condition) | The highest R&D stage reached for the candidate for or any condition or indication. | Free text | e.g. 'Phase II (Snake venom poisoning)' or 'Marketed (Cancer)' | N/A |
| Development status | Whether the candidate's R&D is currently active or inactive | Drop-down (list) | Active Inactive | This is not defined but a time cut-off. Instead, review the available evidence, preclinical data, clinical trial entries etc to determine if a candidate is under active development or not. If no recent activity is evident, or there is clear evidence of their discontinuation since then, mark as inactive. |
| Developers/investigators | List of key organisations involved in the candidate's development. This includes current developers, originators, Universities; patent owners etc. | Free text | e.g. BTG Specialty Pharmaceuticals; Protherics | If the 'originator' is different from developer, but still an existing organisation, include in the developer field as well as current developer. Include all relevant organisations. For organisations in preclinical, include Universities. If off-patent, include 'off-patent'. |
| Known funders | List of key funders (if available) involved in the candidate's development. | Free text | e.g. Wellcome | Only if available. |
| Preclinical results status | Have results been published (any format). | Drop-down (list) | e.g., Results available; Results not available; Unknown; N/A | N/A |
| Type of preclinical results | Nature of publication. Include whichever is the most recent or 'meaningful'. | Drop-down (list) | e.g. Press release; Conference abstract presentation; Preprint article; Peer reviewed article; Government document; Other; N/A | N/A |
| Preclinical results source | URL link to published source, preferably PubMed URL. Include whichever is/are the most recent or meaningful. | Hyperlink | e.g. https://pubmed.ncbi.nlm.nih.gov/34929179/ | PubMed URL if available. |
| Inactive development type | If development status is inactive: A broad classification of reason as to why development of the candidate is inactive | Drop-down (list) | e.g. No recent activity; Safety issues/adverse events; Funding issues; Study design/recruitment/ethics issues; Endpoints not met; Other; N/A | Additional reasons can be added. |
| Inactive development reason | A more detailed reason for why development is inactive. | Free text | e.g. 'R&D terminated because of increased incidence of cardiac arrhythmias or serious adverse events in intervention arm' | N/A |
| Researched in pregnant or lactating women? (if available) | If the candidate has ever included pregnant or lactating women in the study designs | Drop-down (Yes/No) | e.g. Yes; No; Unknown | Not an essential field, but to be filled depending on information availability. |
| If marketed, regulatory approval type/level New field | If the product is already marketed with regulatory approval, what level of approval it has received. | Drop-down (list) - **multiple selections possible** | e.g., WHO prequalification Stringent regulatory approval (SRA) National regulatory approval (NRA) in at least one country Emergency use authorisation | To distinguish levels of marketing approval, which is relevant to the field of SBE R&D. |
| Evidence has been or is tested in clinical trials? | Is there evidence the candidate is either in or has been tested in clinical trials? | Drop-down (Yes/No) | e.g. Yes; No; Unknown | Some marketed medicines/antivenoms have very little associated clinical trial data. This helps capture that. |
| If yes, does clinical trial evidence pre-date 2015? | If there is evidence of clinical trials, were they conducted before the search timespan cut off of 2015? | Drop-down (Yes/No) | e.g. Yes; No; Unknown | Clinical trials before the search timespan cut off should not be included. This function helps filter that. |
| List of clinical trials post-2015 | List of relevant clinical trials post-2015. Automatically filled from CT entries below. | Automated | e.g. https://clinicaltrials.gov/ct2/show/NCT04470791; https://clinicaltrials.gov/ct2/show/NCT00639951 | N/A |
| **REGISTRATION DETAILS (FOR PRODUCTS ONLY)** |  |  |  |  |
| Clinical use status (for SBE) | Whether the candidate is purely investigational/not yet approved, already approved and marketed with stringent or national regulatory approval, marketed and available but with limited regulatory approval (i.e. Emergency Use Authorization only), marketed and available but with no clear regulatory approval, used off-label, or has been withdrawn from the market. | Drop-down (list) | e.g.  Marketed (regulatory approval) Marketed/available (no clear regulatory approval) Investigational / not yet approved Used off-label Withdrawn | This is useful for distinguish some nuances in the SBE R&D landscape, where some antivenoms are fully approved and marketed for use, while others only appear to have limited regulatory approval, such as Emergency Use Authorization, and yet others are marketed and available for SBE but don't have any clear regulatory approval. The idea that some medicines approved for a different conditions are being used off-label already for SBE is not clear, but included here in case. |
| Approval status | Type of approval the products has achieved. | Drop-down (list) | e.g.  approved  conditional approval  approval withdrawn  emergency use authorisation  application under review  approval status unclear | N/A |
| Approving authority | Approval granted by what authority level | Drop-down (list) | e.g.  SRA  NRA  SRA other  WHO prequalification | N/A |
| National Authority Approval status (and date) | Status of approval from relevant authority, and date of approval | Drop-down (list) | e.g.  granted  application under review  application not filed  approval denied  emergency use authorisation  (plus date) | N/A |
| US FDA approval status (and date) | As above | Drop-down (list) | As above | N/A |
| EMA approval status (and date) | As above | Drop-down (list) | As above | N/A |
| Japanese MHLW approval status (and date) | As above | Drop-down (list) | As above | N/A |
| Other stringent Regulatory authority approval (and date) | As above | Drop-down (list) | As above | N/A |
| Stringent Regulatory Authority (SRA) approval (and date) | Status of approval from stringent authority, and date of approval | Drop-down (list) | e.g.  Yes  No  Emergency use authorisation  Article 58  (plus date) | N/A |
| WHO pre-qualification (and date) | Status of WHO pre-qualification, and date of approval | Drop-down (list) | e.g.  Yes  No  Unknown  (and date) | N/A |
| Countries where the product is approved | List of countries where approved | Drop-down (list) | e.g., Ghana; Morocco | N/A |
| Region of use | List of regions where product is used | Drop-down (list) | e.g., North Africa; East Africa; West Africa | Regions where products is *used* not necessarily where it is marketed or effective in (although can be all). |
| Approved for use in pregnant or lactating women | Approval status for pregnant and lactating women, if known. | Drop-down (list) | e.g.  approved for use in pregnant and lactating women  approved for use in pregnant women  approved for use in lactating women  suitable for pregnant and lactating women  not approved for use in pregnant and lactating women  not trailed in pregnant and lactating women  unknown | Not an essential field, but to be filled depending on information availability. |
| FDA pregnancy labelling/pregnancy risk summary | Candidate's FDA pregnancy label (if approved) or pregnancy risk summary (if not yet approved). | Free text | No drug systemic absorption; Risk statement based on human data; Risk statement based on animal data; Risk statement based on pharmacology ; Background risk information in general population; Background risk information in disease population | Information available on FDA drug labels. If not approved (i.e., not available), write 'N/A'. |
| **LINKED CLINICAL TRIAL DATA (IF APPLICABLE)** |  |  |  |  |
| CT title | Exact title as it appears from the source. | Free text | e.g. Study to Evaluate the Efficacy of Two Treatment Schemes With Antivipmyn ® for the Treatment of Snake Bite Envenomation | This may differ slightly between sources. |
| CT number | Unique CT identifier as per CT database. If more than one, list all separated by semi-colons. | Alpha-numeric | e.g. NCT00639951 | If the CT is relevant to more than one candidate in the database, duplicate CT entries should be made, and assigned it to the additional/other candidate/s. |
| CT last updated | CT last updated date as per CT database. Date written as dd/mm/yyyy. | Free text | e.g., 21/03/2021 | If unknown, write 'unknown'. |
| CT phase | CT stage/phase as per CT database. Written exactly as presented. |  | e.g. 'Phase IV' | Write exactly as presented. For those that say 'N/A' or 'unknown', write 'N/A' or 'unknown'. Include specifics such as 'Phase IIb' |
| CT source | Link to clinical trial record. | Free text | [e.g. https://clinicaltrials.gov/ct2/show/NCT04470791](https://clinicaltrials.gov/ct2/show/NCT00639951) | These links can be found in the 'web address' column of the ICTRP data sets. Otherwise the URL where sourced. |
| CT status | CT status as per CT database. | Drop-down (list) | e.g. Terminated (Administrative reasons) | N/A |
| CT terminated type | If CT status is terminated: A broad classification of reason as to why development of the trial was terminated |  | e.g. Funding issues; Study design/recruitment/ethics issues; Endpoints not met; Adverse events; Other; N/A | N/A |
| CT terminated reason | A detailed reason for why the trial was terminated. |  | e.g., 'Clinical trial terminated due to difficulty enrolling suitable candidates within the study period' | N/A |
| CT description | Concise description of CT, in a prescribed format. Information to be included are: the brief study description (can be lifted from CT.gov), the study population, and the intervention – in that order. NO ENDPOINTS are needed. | Free text | e.g. The purpose of this study is to compare whether a same total dose given up front as a single dose is more effective and as safe as the same dose given as a fractioned dose. Evaluate the Utility of the the Dry Tube Test Evaluating its Correlation with Coagulation Test Results (fibrinogen, platelets, INR, PT and PTT). Explore the Evolution of some Serum Markers (CK, DHL, metalloproteinase), Amount of Venom and Antivenom Levels and the Progression of Local Lesion | Copy of the brief description (not the detailed one) exactly as is written from the source. Brief descriptions may be very brief or longer/more descriptive. |
| CT start date | CT start date as per CT database, written as dd/mm/yyyy | Free text | e.g. 01/01/2009 | N/A |
| CT start type | Actual or planned date | Drop-down (list) | Actual | N/A |
| CT end date | CT end date as per CT database, written as dd/mm/yyyy | Free text | e.g.01/03/2009 | Use the "Actual Study Completion Date" |
| CT end date type | Actual or planned date | Drop-down (list) | Actual | N/A |
| CT location(s) | CT location(s) as per CT database. Separated by semi-coloncs | Free text | France; Germany | If unknown, leave blank. |
| CT enrolment | Actual or planned (whichever is most applicable) number of participants to be enrolled in the study. | Numeric | e.g. 150 | If unknown, leave blank. |
| CT results status | Whether CT results been published (any format). | Drop-down (Available/Not available) | e.g., Available; Not available | N/A |
| CT results type | Nature of publication. | Drop-down (list) | e.g. Press release; Conference abstract presentation; Clinical trial registry; Preprint article; Peer reviewed article; Other | N/A |
| CT results source | Link to published source. | Free text | e.g. https://pubmed.ncbi.nlm.nih.gov/33183672/ | Often not listed on CT entries. Searching CT titles can return relevant papers for trials that have completed (time permitting). |
| CT sponsor(s) | CT sponsor(s) as per CT database. Listed separated by semi-colon. | Free text | e.g. Instituto Bioclon S.A. de C.V. | N/A |
| CT collaborators | CT collaborators as per CT database. Listed separated by semi-colon. | Free text | e.g. Universidad Nacional Autonoma de Mexico; University of Arizona | N/A |

1. “Poisoning” was the terminology offered by Adis Insight. [↑](#footnote-ref-1)
