# Supplementary material for: Landscape of toxin-neutralizing therapeutics for snakebite envenoming (2015–2022): Setting the stage for an R&D agenda
Source: PLoS Negl Trop Dis. 2024 Mar 26;18(3):e0012052. doi: 10.1371/journal.pntd.0012052 (PMC10965046; doi:10.1371/journal.pntd.0012052)
Supplement: S2 Text — (DOCX) [file pntd.0012052.s002.docx]

Supplementary material S2.

List of included marketed and/or available products

SBE marketed and/or products

*Biologics*

*Available and approved products*

- *Agkistrodon acutus* antivenom - DaMAV-China (Shanghai Serum Bio-technology Co Ltd, China)
- *Agkistrodon halys* antivenom - AhAV (Shanghai Serum Bio-technology Co Ltd, China)
- Anavip (Instituto Bioclon/Laboratorios Silanes, S. A. de C. V., Mexico)
- Anti Snake Venom Serum Central Africa - 6 (Biological E Limited, India)
- Anti Snake Venom Serum Monovalent *Echis* *ocellatus* (Biological E Limited, India)
- Anti Snake Venom Serum Pan Africa - 10 (Biological E Limited, India)
- Antiveneno Crotálico (Instituto Nacional de Produccion de Biologicos, Argentina)
- Antiveneno *Micrurus* (Instituto Nacional de Produccion de Biologicos, Argentina)
- Anti-Viper antivenom - Russell's viper (Myanmar Pharmaceutical Factory (MPF)/Burma Pharmaceutical Industry (BPI), Myanmar)
- Anti-viper antivenom (Microgen and Ministry of Health, Russian Federation)
- Antiviperine sera (National Center of Infectious and Parasitic Diseases, Bulgaria)
- Antivipmyn Africa (Instituto Bioclon/Laboratorios Silanes, S. A. de C. V., Mexico)
- Antivipmyn TRI Fabotherapic (Instituto Bioclon/Laboratorios Silanes, S. A. de C. V., Mexico)
- ASNA-C (Bharat Serums and Vaccines Limited, India)
- *B. multicinctus* and *B. candidus* antivenom (Vietnam Poison Control Center, Hanoi Medical University)
- Banded krait antivenin, *Bungarus fasciatus* monovalent antivenom - BFMAV (Queen Saovabha Memorial Institute, Thailand)
- BioSave - Serum Anti Bisa Ular Polivalen (PT Bio Farma (Persero), Indonesia)
- Bivalent Haemorrhagic Antivenom - HBAV (CDC, Taiwan)
- Bivalent *Naja*/*Walterinnesia* Snake AV - Equine (National Antivenom & Vaccine Production Center, Saudi Arabia)
- Bivalent Neurotoxic antivenom - FNAV (CDC, Taiwan)
- BothroFAV (MicroPharm Ltd, United Kingdom)
- Bungarus Antivenom (CDC, Taiwan)
- *Bungarus multicinctus* antivenom - BmAV (Shanghai Serum Bio-technology Co Ltd, China)
- Cobra Antivenin (Queen Saovabha Memorial Institute, Thailand)
- CoRal-ICP Liquid (Instituto Clodomiro Picado, Costa Rica)
- Coralmyn (Instituto Bioclon/Laboratorios Silanes, S. A. de C. V., Mexico)
- Crotalidae Polyvalent Immune Fab (ovine) (BTG International Inc., United States of America)
- *Echis Coloratus* Equine Antiserum (Kamada Limited, Israel)
- EchiTAbG (MicroPharm Ltd, United Kingdom)
- EchiTAb-plus-ICP (Instituto Clodomiro Picado, Costa Rica)
- European viper venom antiserum (Imunološki Zavod (Institute of Immunology), Croatia)
- Faboterapico Polivalente Antiviperino (Birmex - Instituto Nacional de Higiene, Mexico)
- Freeze-dried Habu antivenom (KM Biologics Co. Ltd, Japan)
- Freeze-dried Mamushi antivenom (KM Biologics Co. Ltd, Japan)
- Gamma-Vip (Institut Pasteur de Tunis, Tunisia)
- Green Pit Viper Antivenin (Queen Saovabha Memorial Institute, Thailand)
- Haemato-polyvalent snake antivenom - HPAV (Queen Saovabha Memorial Institute, Thailand)
- Hexavalent snake venom immunoglobulin (Razi Vaccine & Serum Research Institute, Iran)
- Indian Snake Anti Venom - I.P (VINS Bioproducts Ltd, India)
- Inoserp MENA (INOSAN BIOPHARMA S. A., Spain)
- Inoserp PAN-AFRICA (INOSAN BIOPHARMA S. A., Spain)
- IPAVIP Antiviperin Sera (Institut Pasteur d'Algerie, Algeria)
- King cobra antivenin (Queen Saovabha Memorial Institute, Thailand)
- Kovax Freeze-Dried *Agkistrodon* (Korean mamushi) Equine Antivenom (KoreaVaccine Co Ltd, Republic of Korea)
- Malayan krait antivenin (Queen Saovabha Memorial Institute, Thailand)
- Malayan Pit Viper Antivenin (Queen Saovabha Memorial Institute, Thailand)
- Monovalent caprine antivenom against *C. rhodostoma* (Twyford Pharmaceutical)
- Monovalent *D. acutus* antivenom - DaMAV-Taiwan (CDC, Taiwan)
- Monovalent *Daboia* *siamensis* antivenom - DsMAV-Taiwan (CDC, Taiwan)
- Monovalent *Naja philippinensis* Cobra Antivenom (Biologicals Manufacturing Division of Research Institute for Tropical Medicine, Philippines)
- Monovalent serum against snake venom gyurza (UzbioPharm LLC, Uzbekistan)
- *Naja atra* monovalent antivenom - NaAV (Shanghai Serum Bio-technology Co Ltd, China)
- Neuro-polyvalent snake antivenom (Queen Saovabha Memorial Institute, Thailand)
- North American Equine Coral Snake Antivenin (Wyeth, USA)
- Pentavalent snake antivenom immunoglobulin (Razi Vaccine & Serum Research Institute, Iran)
- Polisera (Vetal Serum ve Biyolojik Ürünler Üretim Sanayi Tic. A.Ş, Turkey)
- PoliVal-ICP (Instituto Clodomiro Picado, Costa Rica)
- Polyvalent Anti Snake Venom Serum I.P. (King Institute of Preventative Medicine and Research, India)
- Polyvalent Anti-Snake Serum (Egyptian Organisation for Biological Products and Vaccines (VACSERA), Egypt)
- Polyvalent Antisnake Venom Serum (National Institute of Health, Pakistan)
- Polyvalent Anti-Vipers Serum (Egyptian Organisation for Biological Products and Vaccines (VACSERA), Egypt)
- Polyvalent serum against snake venoms gyurza, efa, and cobra (UzbioPharm LLC, Uzbekistan)
- Polyvalent Snake Antivenin I.P. - Asia (Haffkine Biopharmaceutical Corporation Ltd, India)
- Polyvalent Snake Antivenom - Equine (National Antivenom & Vaccine Production Center, Saudi Arabia)
- Russell's viper antivenin (Queen Saovabha Memorial Institute, Thailand)
- SAIMR Boomslang antivenom (South African Vaccine Producers, South Africa)
- SAIMR *Echis* antivenom (South African Vaccine Producers, South Africa)
- SAIMR Polyvalent Snake antivenom (South African Vaccine Producers, South Africa)
- SAV-*Naja* (Institute of Vaccines and Biological Substances, Vietnam)
- SAV-*Trimeresurus* (Institute of Vaccines and Biological Substances, VietNam)
- Seqirus Black Snake antivenom (Seqirus Pty Ltd, Australia)
- Seqirus Brown Snake antivenom (Seqirus Pty Ltd, Australia)
- Seqirus Death Adder antivenom (Seqirus Pty Ltd, Australia)
- Seqirus Polyvalent antivenom (Seqirus Pty Ltd, Australia)
- Seqirus Taipan antivenom (Seqirus Pty Ltd, Australia)
- Seqirus Tiger Snake antivenom (Seqirus Pty Ltd, Australia)
- Siamese cobra antivenin (Myanmar Pharmaceutical Factory, Myanmar)
- SnaFab5 (Padra Serum Alborz, Iran)
- SnaFab6 (Padra Serum Alborz, Iran)
- Snake Antivenin (Polyvalent) IP (Biological E Limited, India)
- Snake venom antiserum - Pan Africa (Premium Serums and Vaccines Pvt. Ltd., India)
- Snake Venom Antiserum African - 10 (VINS-A) (VINS Bioproducts Ltd, India)
- Snake Venom Antiserum Echiven Plus (VINS Bioproducts Ltd, India)
- Snake venom antiserum I.P. (Premium Serums and Vaccines Pvt. Ltd., India)
- Snake Venom Antitoxin - Menaven (VINS Bioproducts Ltd, India)
- Soro Antibotrópico Pentavalente (Instituto Butantan, FUNED, Instituto Vital Brazil, CPPI, Brazil)
- Soro Antibotrópico pentavalente e Crotálico (Instituto Butantan, FUNED, Instituto Vital Brazil, Brazil)
- Soro Antibotrópico pentavalente e Laquético (Instituto Butantan, FUNED, Instituto Vital Brazil, Brazil)
- Soro Anticrotálico (Instituto Butantan, FUNED, Instituto Vital Brazil, CPPI, Brazil)
- Soro Antielapídico Bivalente (Fundacao Ezequiel Dias & Butantan Institute, Brazil)
- Suero Antibotrópico Polivalente (Instituto Nacional de Salud, Peru)
- Suero Anticoral Polivalente (Instituto Nacional de Salud, Colombia)
- Suero Anticrotálico Monovalente (Instituto Nacional de Salud, Peru)
- Suero Antilachésico Monovalente (Instituto Nacional de Salud, Peru)
- Suero Antiofídico (BIOTECFAR, Venezuela)
- Suero Antiofídico Anticoral Polivalente Liofilizado (Laboratorios Biologicos PROBIOL Ltda, Colombia)
- Suero Antiofidico Polivalente (Instituto Nacional de Salud, Colombia)
- Suero Antiofidico Polivalente BIOL (Instituto Biologico Argentino S.A.I.C, Argentina)
- Suero Antiofidico Polivalente Botropico/Crotalico (Ministerio de Salud y Deportes, Instituto Nacional de Laboratorios De Salud, Bolivia)
- Suero Antiofidico Polivalente Botropico/Laquesico (Ministerio de Salud y Deportes, Instituto Nacional de Laboratorios De Salud, Bolivia)
- Suero Antiofidico Polivalente Centroamericano BIOL CLB (Instituto Biologico Argentino S.A.I.C, Argentina)
- Suero Antiofidico Polivalente Liofilizado (Laboratorios Biologicos PROBIOL Ltda, Colombia)
- Suero Bothropico Bivalente (Instituto Nacional de Produccion de Biologicos, Argentina)
- Suero Bothropico Tetravalente (Instituto Nacional de Produccion de Biologicos, Argentina)
- Viekvin (Institute of Virology, Vaccine and Sera, TORLAK, Serbia)
- Viper venom antitoxin (BIOMED Wytwornia Surowic i Szczepionek, Poland)
- *Vipera palaestinae* Equine Antiserum (Kamada Limited, Israel)
- ViperaTAb (MicroPharm Ltd, United Kingdom)
- VIPERFAV (MicroPharm Ltd, United Kingdom)

*Approved – discontinued products*

- FAV-Afrique (Sanofi Pasteur, MicroPharm)
- Favirept (MicroPharm UK, previously Sanofi-Pasteur, France)

*Unclear approval pathway*

- Anti-*Yamakagashi* Antivenom (KM Biologics Co. Ltd, Japan)
- *Bungarus candidus* Antivenom (Venom Research Unit, University of Medicine and Pharmacy Ho Chi Minh City, Vietnam)
- *Bungarus candidus* Monospecific Antivenom (Venom Research & Antivenom Production Unit, National Poison Control Center, Vietnam)
- *Calloselasma rhodostoma* - Malayan Pit Viper Antivenom (Venom Research Unit, University of Medicine and Pharmacy Ho Chi Minh City, Vietnam)
- Combipack of Snake Venom Antiserum (African- Ten) (Premium Serums and Vaccines Pvt. Ltd., India)
- *Daboia Russelii* Mono (VINS Bioproducts Ltd, India)
- *Naja kaouthia* Antivenom (Venom Research Unit, University of Medicine and Pharmacy Ho Chi Minh City, Vietnam)
- *Naja Kouthia* Mono (VINS Bioproducts Ltd, India)
- *Naja siamensis* Antivenom (Venom Research Unit, University of Medicine and Pharmacy Ho Chi Minh City, Vietnam)
- *Ophiophagus hannah* Antivenom (Venom Research Unit, University of Medicine and Pharmacy Ho Chi Minh City, Vietnam)
- Polyvalent Big Four Fab'2 antivenom (Incepta Vaccines, Bangladesh)
- SIIPL-01 Polyvalent Anti-snake Venom Serum (Serum Institute, India)
- Snake Venom Antiserum - Central Africa (Premium Serums and Vaccines Pvt. Ltd., India)
- Snake Venom Antiserum Afriven (VINS Bioproducts Ltd, India)
- Snake Venom Antiserum *Echis Ocellatus* - Echiven (VINS Bioproducts Ltd, India)
- V-ASV polyvalent antivenom (Virchow Biotech, India)
