# Supplementary material for: Landscape of toxin-neutralizing therapeutics for snakebite envenoming (2015–2022): Setting the stage for an R&D agenda
Source: PLoS Negl Trop Dis. 2024 Mar 26;18(3):e0012052. doi: 10.1371/journal.pntd.0012052 (PMC10965046; doi:10.1371/journal.pntd.0012052)
Supplement: S3 Text — (DOCX) [file pntd.0012052.s003.docx]

Supplementary material S3.

List of included investigational candidates

Investigational SBE candidates

*Biologics*

*Immunoglobulin products - animal plasma/serum derived*

- Chicken anti-neurotoxin IgY (ANT- IgY) (egg yolk derived) (against cobra and krait)
- Chicken IgY (egg yolk derived) (against *Bitis arietans* and *Crotalus durissus terrificus*)
- Chicken IgY (egg yolk derived) (against *Bothrops alternatus*)
- Chicken IgY (egg yolk derived) (against *Bungarus multicinctus*)
- Chicken IgY (egg yolk derived) (against Cobra, Krait, Russells Viper and Saw-scaled Viper)
- Chicken IgY (egg yolk derived) (against *D. acutus*, China)
- Chicken IgY (egg yolk derived) (against *D. acutus*, Taiwan)
- Chicken IgY (egg yolk derived) (against *Daboia russelii formosensis*)
- Chicken IgY (egg yolk derived) (against *Naja naja atra*)
- Chicken IgY (egg yolk derived) (against Oxyuranus *scutellatus*)
- Chicken IgY (egg yolk derived) (against *Trimeresurus albolabris*)
- Chicken IgY (egg yolk derived) (against *Trimeresurus mucrosquamatus*)
- Chicken IgY (egg yolk derived) (against *Trimeresurus stejnegeri*)
- Combined *Bothrops* AV + synthetic SVSP peptides pepB and pepC
- Inoserp Europe polyvalent antivenom (against European vipers)
- Murine monoclonal 3FTx-specific IgGs (against *Naja ashei*)
- Novel anti-*Crotalus mictlantecuhtli* rabbit antiserum (against *C. mictlantecuhtli*)
- Novel anti-crotamine polyclonal antibodies (against *Crotalus molossus nigrescens*)
- Novel anti-crotamine polyclonal antibodies (against *Crotalus oreganus helleri*)
- Novel anti-crotamine polyclonal antibodies via recombinant fusion protein immunization (against *Crotalus* spp)
- Novel anti-short-chain α-neurotoxin (ScNtx) antivenom via toxin immunization (against elapids)
- Novel anti-short-chain α-neurotoxin D.H. rabbit antiera via toxin immunization (against *Micrurus diastema*)
- Novel anti-SVSP antivenom via toxin immunization (against *Bothrops jararaca*)
- Novel bivalent snake antivenom (IgG) (against *﻿Daboia russelii* & *Echis carinatus*) (Pakistan)
- Novel camelid IgG antivenom (against *Echis sochureki*)
- Novel equine anti-*Bitis* antivenom (against *B.* *arietans*)
- Novel equine anti-*Bitis* antivenom (against *B. nasicornis* and *B. rhinoceros*)
- Novel equine anti-elapid antivenom (against *N. annulifera*, *D. polylepis*, *D. angusticeps*)
- Novel equine anti-*Naja* antivenom (against *N. melanoleuca*)
- Novel equine anti-*Naja* antivenom (against *N. mossambica*)
- Novel equine blended anti-*Micrurus tener* and anti-ScNtx antibodies (against elapids)
- Novel equine F(ab')2 antivenom via streamlined processing (against *Vipera ammodytes*)
- Novel equine pan-specific antiserum via diverse-toxin immunization (against elapids)
- Novel equine/rabbit broad-spectrum antiserum via r3FTX toxin immunization (against cobra spp)
- Novel F(ab')2 antivenom (against *Daboia russelii siamensis*) (China)
- Novel freeze-dried trivalent antivenom (FDTAV) (against *Bothrops, Lachesis, Crotalus*)
- Novel ICP-AVRI-UOP Sri Lankan polyspecific antivenom
- Novel murine anti-haemorrhagic antivenom via DNA immunization (against *Echis ocellatus*)
- Novel murine antiserum via DNA+ protein boost immunization (against *Micrurus corallinus*)
- Novel murine antivenom via toxin/peptide immunization (against *Deinagkistrodon acutus*)
- Novel ovine pathology-specific experimental antivenom (EAV) 1 (against VICC/haemotoxic)
- Novel ovine pathology-specific experimental antivenom (EAV) 2 (against VICC/haemotoxic)
- Novel pan-specific antivenom (against medically significant snakes of India) (Project)
- Novel PNG taipan antivenom (against *Oxyuranus* *scutellatus*)
- Novel polyvalent equine antivenom (against *Micrurus* spp, Argentina)
- Novel polyvalent equine antivenom (against *Micrurus* spp, Colombia)
- Novel polyvalent murine, equine and rabbit antisera (against *Micrurus* spp, Brazil)
- Novel rabbit antivenom via venom plus toxin immunization (against *Micrurus* spp)
- Novel Sri Lankan Polyvalent Antivenom (SL PAV)
- Rabbit anti-rDisintegrin polyclonal antibodies (ARDPAs) via recombinant toxin immunization (against *Crotulus* spp)
- Snake (*Micrurus*) North American immune F(ab')2 Equine

*Immunoglobulin products - recombinant*

- Broadly neutralizing antibodies (against Indian and African snakes) (Project)
- Broadly Neutralizing svMP-specific Human mAbs (against North American vipers) (Project)
- Camelid nanobodies (VHH and VHH-Fc) (plant expressed) (against *Naja kaouthia*/α-cobratoxin)
- Camelid nanobodies (VHH) (against *Bothrops atrox*)
- Camelid nanobodies (VHH) (against *Bothrops jararacussu*)
- Camelid nanobodies (VHH) (against Cobra toxin) (Project)
- Camelid nanobodies (VHH) (against *Daboia russelii*)
- Camelid nanobodies (VHH) (against necrosis-inducing venom toxins (NITs)) (Project)
- Chicken scFv (egg yolk derived) (against *Bungarus multicinctus*)
- Chicken scFv (egg yolk derived) (against *D. acutus*, Taiwan)
- Chicken scFv (egg yolk derived) (against *Daboia russelii formosensis*)
- Chicken scFv (egg yolk derived) (against *Naja naja atra*)
- Chicken scFv (egg yolk derived) (against *Trimeresurus mucrosquamatus*)
- Chicken scFv (egg yolk derived) (against *Trimeresurus stejnegeri*)
- Combined humanised IgG and camelid VHHs antivenom for Sub-Saharan Africa (Project)
- Human anti-kaouthiagin scFv (15, 20, and 61) (against *Naja* *kaouthia*)
- Human monoclonal antibodies (IgG) (broad spectrum anti-snake venom) (Project)
- Human oligoclonal recombinant IgG antibodies (against *Dendroaspis polylepis*)
- Human polyclonal scFv (against multiple Iranian snakes)
- Human recombinant IgG antibodies (against *Naja kaouthia*/α-cobratoxin)
- Human recombinant polyclonal F(ab) (against *Echis* *carinatus*)
- Human scFv (against *Macrovipera lebetina*)
- Human scFv (B7, C11, and E9) (against *Bothrops* *jararacussu* and *Crotalus durissus terrificus*)
- Human scFv (C13, C24, C39, C43, and C45) (against *Naja oxiana*)
- Human scFv (G12F3) (against *Naja oxiana*)
- Humanised murine mAbs (against venom-induced consumption coagulopathy) (Project)
- PEO-1 plantivenom (camelid VHH, plant expressed) (against *Bothrops* *asper*)
- scFvBaP1 (plant expressed) (against *Bothrops asper*)
- scFv-Svmp (chicken derived, plant expressed) (against *Bothrops pauloensis*)
- Vipax (synthetically evolved camelid nanobody-based antivenom)

*Non-immunoglobulin products - animal/naturally derived/recombinant*

- BJ46a (endogenous SVMPI) (against *Bothrops jararaca*)
- Naked DNA (Calf thymus)
- rAnti-3FTX nAChR-binding proteins (Ls-AChBP and humanized α7-AChBP)
- rBaltMIP (alpha snake blood PLA_2_ inhibitor) (from *Bothrops alternatus*)
- rDM64 / DM64 protein (from opossum protein DM64)
- Recombinant endogenous snake toxin inhibitors (Project)
- rLTNF-11 peptide (from opossum protein oprin)
- rOprin-like (DM43-like) protein (from opossum protein oprin/DM43)
- rTryptase β / Tryptase β (human mast cell tryptase)
- saPLIγ (gamma snake blood PLA_2_ inhibitor) (from *Sinonatrix annularis*)

*Drugs*

*Therapeutic - natural/botanical*

- 14-acetylandrographolide (Andrographis paniculata extract isolate)
- 14-deoxy-11,12 didehydroandrographolide (Andrographis paniculata extract isolate)
- 14-deoxy-11-oxoandrographolide (Andrographis paniculata extract isolate)
- 2-hydroxy-4-methoxybenzaldehyde (polyphenol plant extract isolates)
- 4',7-dihydroxy-5-methoxyflavone-8-C-β-D-glucopyranoside (Oxalis corniculata extract isolate)
- Andrograpanin (Andrographis paniculata extract isolate)
- Aristolochic acid (Artistolochia sp. extract isolate)
- Bakuchiol (plant extract isolate)
- Betulinic acid
- BRS-P19 (Bauhinia rufescens seed extract isolate)
- Butein (plant extract isolate)
- Caffeic acid (polyphenol plant extract isolate)
- Caftaric acid (polyphenol plant extract isolate)
- Casuarictin (Laguncularia racemosa extract isolate)
- Chicoric acid (polyphenol plant extract isolate)
- Chlorogenic acid (polyphenol plant extract isolate)
- Crepiside E beta glucopyranoside (Elephantopus scaber extract isolate)
- Fucoidan (Brown seaweed extract isolate)
- Gallic acid (polyphenol plant extract isolate)
- Go3 (Green seaweed extract isolate)
- Hesperetin (citrus extract isolate)
- Hispidulin (Moquiniastrum floribundum/Aegiphila integrifolia extract isolate)
- Ikshusterol3-O-glucoside (Clematis gouriana extract isolate)
- Isoandrographolide (Andrographis paniculata extract isolate)
- Jatromollistatin (Jatropha mollissima extract isolate)
- Kolaviron (flavanoid plant extract isolate)
- Lansiumamide B (Clausena excavata extract isolate)
- Lupeol (Aegiphila integrifolia extract isolate)
- Mannitol (Aegiphila integrifolia extract isolate)
- Mimosine (Mimosa pudica extract isolate)
- Myricetin (polyphenol plant extract isolate)
- Oleanolic acid
- p-Coumaric acid (polyphenol plant extract isolate)
- Pectolinarigenin (Aegiphila integrifolia extract isolate)
- Pinostrobin (Renealmia alpinia extract isolate)
- Piperine (Piper longum L extract isolate)
- Quercetin (polyphenol plant extract isolate)
- Quercitrin (Euphorbia hirta/polyphenol extract isolate)
- Rosmarinic acid (polyphenol plant extract isolate)
- Rutin/Rutin succinate (polyphenol plant extract isolate)
- Scutellarin (flavanoid plant extract isolate)
- Silymarin (milk thistle extract isolate)
- Spiro [androst-5-ene-17,1'-cyclobutan]-2'-one,3-hydroxy-(3β,17β)
- Stigmasterol (Aegiphila integrifolia extract isolate)
- Sulfated agaran (Red seaweed extract isolate)
- Tannic acid (polyphenol plant extract isolate)
- Ursolic acid
- Vanillic acid (polyphenol plant extract isolate)
- Vitamin B complex
- Vitamin C (Ascorbic acid)
- Vitamin E
- Vitexin (flavanoid plant extract isolate)
- Zinc / zinc oxide (ZnO) complex (ZC)
- β-sitosterol (Aegiphila integrifolia/citrus extract isolate)

*Therapeutic – synthetic*

- 1-(2-methyl-8-naphthalen-1-yl-imidazo-[1,2-α]pyridin-3-yl)ethanone
- 4-(2-aminoethyl) benzenesulfonyl fluoride hydrochloride (AEBSF)
- 4-benzoyl-3-hydroxyphenyl benzoate (BHB)
- Abiotic hydrogel nanoparticle (against Elapids)
- Abiotic synthetic nanoparticle TIMP-mimicking polymers (against SVMPs)
- Acetylsalicylic acid (ASA)
- ADDovenom: ADDomer and ADDobody protein-based NP neutralizing superbinders (Project)
- Anti-batroxobin (SVSPIs) peptides (pepC and pepB) (against *Bothrops jararaca*)
- Anti-dendrotoxin peptides (via phage display) (against *Dendroaspis polylepis*)
- Anti-myotoxin II peptides (via phage display) (against *Bothrops asper*)
- Anti-necrotic enzyme inhibitors (against necrosis-inducing venom toxins) (Project)
- Anti-PLA_2_ peptides (via phase display/on M13 phages) (against Western cottonmouth)
- Anti-α-cobratoxin peptides (via phage display) (against *Naja kaouthia*)
- Batimastat
- C60 fullerene nanoparticle
- Carbodithioates (benzyl 4-nitrobenzenecarbodithioate)
- Dexketoprofen
- Diethylene triamine pentaacetic acid (DTPA)
- Dimercaprol
- Disulfiram
- DMPS (Unithiol)
- DNA aptamers (against *Bungarus multicinctus*/α-bungarotoxin)
- DNA aptamers (against Daboxin P/*Daboia russelii*)
- DNA aptamers (against *Naja melanoleuca*)
- EDTA / CaNa2EDTA
- Flurbiprofen
- Gold nanoparticle conjugated andrographolide (GNC-andrographolide)
- Gold nanoparticle Vitex negundo conjugated (VN-GNP)
- Gold nanoparticle-conjugated 2-hydroxy-4-methoxybenzoic acid (GNP-HMBA)
- Heparin / LMWH
- Ketoprofen
- Marimastat
- Marimastat Varespladib mixture
- Methyl-varespladib
- Morphine
- N,N,N',N'-tetrakis (2-pyridylmethyl) ethane-1,2-diamine (TPEN)
- Nafamostat
- Oral broad small molecule toxin inhibitors (Project)
- p-bromophenacyl bromide (pBPB)
- Prinomastat
- Silver nanoparticles (AgNPs)
- Sodium silicate complex (SSC)
- Suramin
- Synthetic peptides from atPLIγ (gamma snake blood PLA_2_ inhibitor) (from *Bothrops atrox*)
- Synthetic SVMPI peptides: pERW and pEKW (against *Daboia russelii siamensis*)
- Synthetic variant peptide BLG-col (from β-Lactoglobulin, Buffalo Colostrum)
- Thioesters (2-Sulfenyl Ethylacetate derived)
- Thiosemicarbazones (5A and 5B)
- Titanium dioxide nanoparticles (TiO2-NPs)
- Varespladib
- X-Aptamers (against North American snakes) (Project)
